# Patritumab deruxtecan in leptomeningeal metastatic disease of solid tumors: the phase 2 TUXEDO-3 trial

**DOI:** 10.1038/s41591-025-03744-1

**Published:** 2025-05-30

**Authors:** Matthias Preusser, Javier Garde-Noguera, Juan José García-Mosquera, María Gion, Richard Greil, Miriam Arumi, Manuel Ruiz-Borrego, Antonio Llombart-Cussac, María Valero, Javier Cortés, Marta Campolier, José Antonio Guerrero, Paula González-Alonso, Carlos Jiménez-Cortegana, Jose Rodríguez-Morató, Marta Vaz-Batista, Felicitas Oberndorfer, Maximilian Marhold, Anna Sophie Berghoff, Julia Furtner, Thorsten Fuereder, Rupert Bartsch

**Affiliations:** 1 Department of Medicine 1, Division of Oncology, Medical University of Vienna, Vienna, Austria; 2 Hospital Arnau de Vilanova, Valencia, Spain; 3 Departamento de Medicina, Facultad de Ciencias de la Salud, Universidad Cardenal Herrera-CEU, Alfara del Patriarca, Valencia, Spain; 4 Dr. Rosell Oncology Institute (IOR), Dexeus University Hospital, Pangaea Oncology, Quironsalud Group, Barcelona, Spain; 5 Ramón y Cajal University Hospital, Madrid, Spain; 6 IOB Madrid, Hospital Beata María Ana, Madrid, Spain; 7 IIIrd Medical Department, Paracelsus Medical University Salzburg; Salzburg Cancer Research Institute-Center for Clinical Cancer and Immunology Trials (SCRI-CCCIT); Cancer Cluster Salzburg, Salzburg, Austria; 8 Vall d’Hebron Instituto de Oncología, Barcelona, Spain; 9 Hospital Universitario Virgen del Rocío, Sevilla, Spain; 10 Medica Scientia Innovation Research (MEDSIR), Barcelona, Spain; 11 Hospital Quirónsalud Sagrado Corazón, Sevilla, Spain; 12 International Breast Cancer Center (IBCC), Pangaea Oncology, Quiron Group, Barcelona, Spain; 13 Department of Medicine, Universidad Europea de Madrid, Faculty of Biomedical and Health Sciences, Madrid, Spain; 14 Oncology Department, Hospital Universitario Torrejón, Ribera Group, Madrid, Spain; 15 Hospital Professor Doutor Fernando Fonseca EPE, Lisbon, Portugal; 16 Department of Pathology, Medical University of Vienna, Vienna, Austria; 17 Research Center for Medical Image Analysis and Artificial Intelligence (MIAAI), Faculty of Medicine and Dentistry, Danube Private University, Krems, Austria

**Keywords:** Metastasis, Cancer therapy

## Abstract

Leptomeningeal metastatic disease (LMD) is a severe complication of solid cancers with poor outcomes and limited treatment options. The antibody–drug conjugate patritumab deruxtecan (HER3-DXd) demonstrated efficacy in breast and lung cancers, and HER3 is involved in central nervous system metastases, particularly in parenchymal colonization. In this study, we investigated HER3-DXd efficacy and safety in patients with LMD in cohort 3 of the TUXEDO-3 phase 2 trial. Key eligibility criteria included age ≥18 years, treatment-naive LMD or LMD progressing after radiotherapy from any solid tumor and Eastern Cooperative Oncology Group performance status of 0–2. Between January and July 2024, 20 evaluable patients (nine with type I and 11 with type II LMD) were accrued and received HER3-DXd 5.6 mg kg^−1^ intravenously every 3 weeks. Main primary tumor types included breast (60%) and lung (30%) cancers. Median follow-up time was 5.4 months. The primary endpoint was met with 65.0% patients alive after 3 months. The Kaplan–Meier-estimated 3-month and 6-month overall survival rates were 69.6% and 58.9%, respectively. Overall response rate was 11.1% for intracranial, 30.8% for extracranial and 26.3% for overall lesions. Clinical benefit rate was 50.0% for intracranial, 38.5% for extracranial and 47.4% for overall lesions. Neurological symptoms and quality of life remained stable or improved during study treatment. No new neurological adverse events were observed. The most common adverse events of any grade were anemia (nine (40.9%) patients, one (4.5%) grade ≥3), nausea (seven (31.8%) patients, no grade ≥3), neutropenia (six (27.3%) patients, three (13.6%) grade ≥3), diarrhea (six (27.3%) patients, one (4.5%) grade ≥3), asthenia (six (27.3%) patients, no grade ≥3) and thrombocytopenia and headache (five (22.7%) patients, one (4.5%) grade ≥3 each). TUXEDO-3 showed clinically relevant HER3-DXd activity in patients with LMD. ClinicalTrials.gov identifier: NCT05865990.

## Abstract (web summary)

As presented at the 2025 ASCO Annual Meeting: in cohort 3 of the phase 2 TUXEDO-3 trial, patients with leptomeningeal metastatic disease from any solid tumor were treated with the HER3-targeting antibody–drug conjugate patritumab deruxtecan and showed encouraging 3-month overall survival rates.

## Main

Leptomeningeal metastatic disease (LMD) is defined as the spread of cancer cells to the leptomeninges or the cerebrospinal fluid (CSF) in the subarachnoid space^1,2^. LMD has been reported to occur in up to 10% of patients with solid cancers, most commonly in lung cancer, breast cancer and melanoma. LMD is associated with high morbidity and may cause debilitating neurological symptoms, such as headache, radicular pain, nausea, gait difficulties, cranial nerve palsies, radicular signs and others. Prognosis is poor, with median overall survival (OS) reported across tumor types ranging from 4 weeks to 6 weeks in untreated patients and from 2 months to 6 months in patients responding to currently available treatments. Therapeutic options are limited and based mainly on low level of evidence, as patients with LMD are systematically excluded from clinical trials, and only few prospective studies specifically enrolled patients with LMD^1,2^. In addition, the incidence of LMD among patients with breast cancer has increased due to OS improvements after using chemotherapy and targeted therapies with poor central nervous system (CNS) penetration^3^. The main treatment options recommended in clinical practice guidelines include radiotherapy, intrathecal or intravenous pharmacotherapy and palliative care, with treatment algorithms considering patient performance status, type of LMD (type I, which requires positive CSF cytology or positive LMD biopsy, and type II, which requires only clinical findings and neuroimaging^4,5^), primary tumor type and molecular subtype and prior therapies^1,2,5^. To date, the prognosis remains dismal, and novel treatment opportunities are urgently needed for patients with LMD^2^.

Human epidermal growth factor receptor 3 (HER3/ErbB3) belongs to a family of receptor tyrosine kinases with oncogenic properties^6,7^. Binding of HER3 to its ligands heregulin or NRG-2 leads to a change in its conformation, which facilitates heterodimerization with other ErbB family members, most importantly HER2, and activation of signal transduction. Through this mechanism, HER3 activation promotes tumor growth, proliferation, invasion, metastasis and chemotherapy resistance. HER3 expression has been observed in several tumor types, such as lung cancer, breast cancer, melanoma and others^8,9^. HER3 has recurrently been described to facilitate CNS colonization by cancer cells^10–13^. We previously showed frequent overexpression of HER3 in 75% and 73% of brain metastases (BMs) of breast cancer and non-small-cell lung cancer (NSCLC), respectively^14^. Of note, HER3 expression was more frequent in CNS metastases than in extracranial tumor sites in patients with NSCLC^12,14^.

Patritumab deruxtecan (HER3-DXd) is an antibody–drug conjugate (ADC) developed for intravenous application and consisting of a fully human anti-HER3 IgG1 antibody attached to topoisomerase I inhibitor payloads via a tetrapeptide-based cleavable linker with a drug-to-antibody ratio of 8:1. HER3-DXd has shown a manageable safety profile characterized by a treatment-related discontinuation rate due to adverse events (AEs) of approximately 10%, gastrointestinal and hematologic toxicities as the most common treatment-emergent adverse events (TEAEs) and evidence of relevant anti-tumor activity in clinical trials in EGFR-mutated NSCLC and breast cancer^15,16^. The clinical development of HER3-DXd is ongoing, with several clinical trials evaluating its efficacy, including the phase 3 HERTHENA-Lung02 (ref. ^17^), the phase 2 HERTHENA-PanTumor01 (ref. ^18^) and the phase 2 HERTHENA-Breast01 (ref. ^19^) trials.

Although it was widely thought that ADCs are large and complex molecules that do not readily cross the intact blood–brain barrier^20^, substantial and clinically relevant activity of ADC has been documented in BMs and also LMD^21^. Based on high intracranial response rates against parenchymal BMs seen in several studies, including the TUXEDO-1 (NCT04752059), DEBBRAH (NCT04420598) and DESTINY-Breast12 (NCT04739761) trials^22–25^, the HER2-targeting ADC trastuzumab deruxtecan (T-DXd) is recommended for therapy of patients with active BMs of HER2-positive breast cancer in contemporary clinical practice guidelines^26^. Small studies have also indicated favorable activity of intravenous T-DXd monotherapy in patients with breast cancer with LMD^27,28^. For HER3-DXd, preliminary evidence of CNS activity was observed in the phase 2 HERTHENA-Lung01 trial (NCT04619004) enrolling patients with advanced EGFR-mutated NSCLC. Among 30 of 225 patients accrued to this trial with non-irradiated parenchymal BMs at baseline, an intracranial objective response rate of 33.3% (95% confidence interval (CI), 17.3–52.8) was observed and confirmed per central radiology review^15^. To our knowledge, data on the activity of HER3-DXd in patients with LMD are not available so far.

Given the high unmet clinical need in patients with CNS metastases, established intracranial activity of T-DXd, the biological implication of HER3 overexpression in CNS tumors and the preliminary evidence of CNS activity of HER3-DXd, we performed the prospective, international, multicenter, single-arm phase 2 TUXEDO-3 trial (NCT05865990) to investigate CNS activity of HER3-DXd in active breast and lung cancer BMs and LMD of various tumor types. Here we report the results of cohort 3, which was specifically designed to prospectively evaluate the efficacy and safety of HER3-DXd in patients with LMD from any solid tumor.

## Results

### Patient characteristics

Between 25 January 2024 and 2 July 2024, a total of 20 patients (18 female and two male) with newly diagnosed and untreated LMD from any advanced solid tumor met all the inclusion criteria and were enrolled from seven sites across Austria and Spain. According to the eligibility criteria, patients with progressing LMD after radiotherapy were also allowed; however, there were no cases of progressing LMD during the enrollment process, and patients with newly diagnosed LMD were finally included in the study. Of the enrolled patients, nine (45%) had type I and 11 (55%) had type II LMD, and 14 (70%) had advanced disease at diagnosis. The primary tumor locations were breast in 12 (60%) patients, lung in six (30%) patients, melanoma in one (5%) patient and ovary in one (5%) patient. The median number of previous treatment lines in advanced disease was two (range, 0–6). Previous treatment included ADCs (35%), tyrosine kinase inhibitors (TKIs) (20%) or chemotherapy combined with immunotherapy (20%). The median age at inclusion was 51.5 years (range, 40–66); Eastern Cooperative Oncology Group performance status (ECOG PS) was 0 in six (30%) patients; and 12 (60%) patients had neurological symptoms at baseline. Two (10%) patients did not have evaluable CNS lesions at baseline, and seven (35%) patients did not have evaluable extracranial lesions at baseline. Twelve patients (60%) had visceral disease, and six (30%) had brain-only disease. Patient characteristics are summarized in Table 1.

**Table 1:** Patient characteristics at baseline

Characteristic	*n* (%)
Sex	
Female	18 (90)
Male	2 (10)
Age: median (min; max)	
Age at baseline (years)	51.5 (40.0; 66.0)
ECOG PS	
0	6 (30)
1	9 (45)
2	5 (25)
Primary tumor location	
Breast	12 (60)
HER2	2 (10)
Luminal	4 (20)
Triple-negative	6 (30)
Lung	6 (30)
Ovary	1 (5)
Melanoma	1 (5)
LMD type	
Type I	9 (45)
Type II	11 (55)
Status of LMD	
Progressing after local therapy	0 (0)
Untreated	20 (100)
Advanced disease at diagnosis	
Yes	14 (70)
No	6 (30)
Visceral disease	
Yes	12 (60)
No	7 (35)
No lesions at baseline	1 (5)
Brain-only disease	
Yes	6 (30)
No	13 (65)
No lesions at baseline	1 (5)
Bone or liver metastases	
Yes	10 (50)
Bone metastases	6 (30)
Liver metastases	9 (45)
No	10 (50)
Neurological symptoms at baseline	
Yes	12 (60)
No	8 (40)
Previous ADCs	
Yes	7 (35)
Trastuzumab deruxtecan	1 (5)
Sacituzumab govitecan	5 (25)
Mivertuximab soravtansine	1 (5)
No	13 (65)
Previous tucatinib	
Yes	0 (0)
No	20 (100)
Previous chemotherapy + immunotherapy	
Yes	4 (20)
No	16 (80)
Previous TKIs	
Yes	4 (20)
Osimertinib	3 (15)
Tepotinib	1 (5)
Crizotinib	1 (5)
Sotorasib	1(5)
No	16 (80)
Previous treatment lines in advanced disease: median (min; max)	2 (0; 6)

**Fig. 1:**
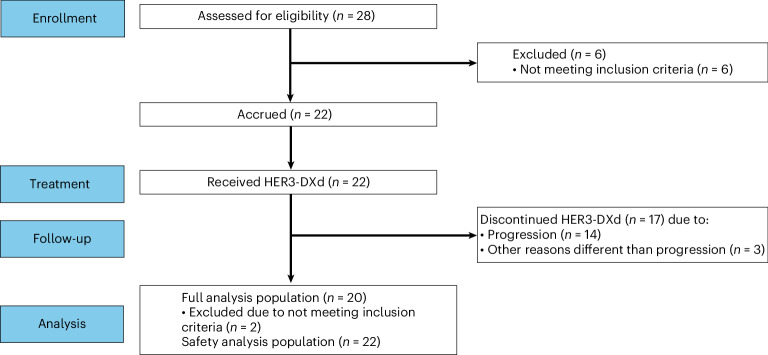
CONSORT flow diagram. Flow-chart showing the number of patients in cohort 3 of TUXEDO-3 who were enrolled, treated, followed-up and included for analysis.

Two additional female patients also received at least one cycle of HER3-DXd but had to be replaced because medical monitoring revealed that one specific inclusion criterion had not been met at enrollment (both patients had received previous systemic treatment for LMD before enrollment) (Supplementary Table 1). As described in the Clinical Study Protocol and the Statistical Analysis Plan, these patients were excluded from the intention-to-treat (ITT) population but were included in the safety analyses (*n* = 22). At data cutoff (28 February 2025) and a median follow-up time of 5.4 months (range, 0.8–12.0), four (20%) patients were still on treatment. The main reasons for treatment discontinuation were death due to clinical progressive disease in six (30%) patients, but with no evidence of radiological progression, and radiologically proven progression of disease in seven (35%) patients. Reasons for treatment termination were progressive disease (50%) and patient’s decision (5%). A CONSORT diagram is provided in Fig. 1.

### Primary outcome analysis

In the first stage of this trial (stage I, enrollment between 25 January 2024 and 8 May 2024), four out of the 10 planned participants were alive 3 months after treatment initiation, meeting the predefined threshold for the interim analysis. Consequently, the study progressed to the second stage, with the accrual of 10 additional patients (stage II enrollment, concluding on 2 July 2024).

**Fig. 2:**
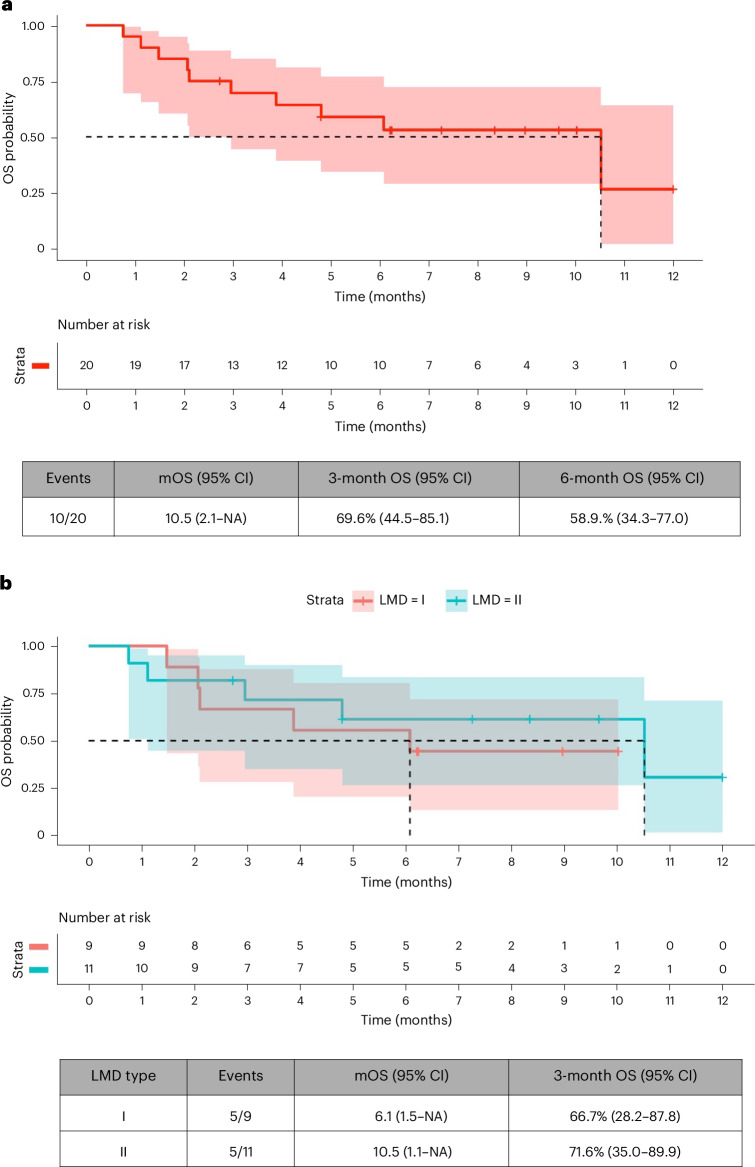
Kaplan–Meier OS curves. Curves are shown for all patients with LMD (*n* = 20) (**a**) and for patients stratified by LMD subtype (**b**). Shaded areas represent 95% CI. mOS, median overall survival.

**Extended Data Fig. 1:**
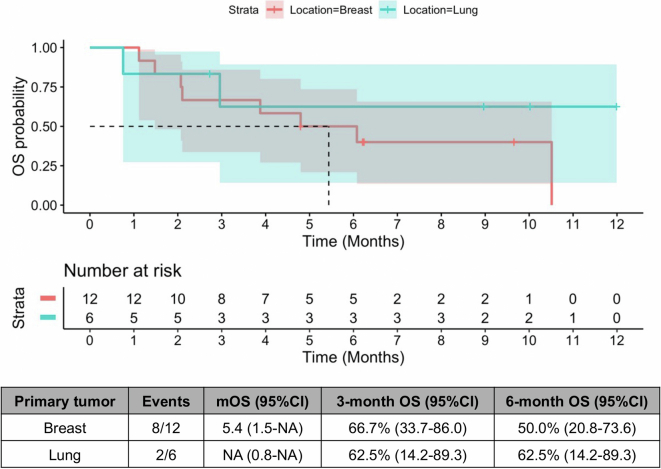
Kaplan-Meier overall survival curve for study participants in patients with LMD from breast and lung cancers. Shaded areas represent 95%CI. CI: Confidence Intervals, LMD: Leptomeningeal disease, mOS: Median overall survival, NA: Not achieved, OS: Overall survival.

At data cutoff (28 February 2025), median treatment duration was 3.4 months (range, 0.0–11.5). Thirteen out of 20 patients were alive after 3 months of treatment initiation, and the 3-month OS rate was 65%, meeting the primary objective of the study. The primary tumors of these 13 patients were breast cancer in eight (61.5%), lung cancer in three (23.1%), melanoma in one (7.7%) and ovarian cancer in one (7.7%), and the 3-month OS rate by tumor type was 66.7% (8/12) in breast cancer, 50% (3/6) in lung cancer, 100% (1/1) in ovary cancer and 100% (1/1) in melanoma. The Kaplan–Meier-estimated 3-month OS rate was 69.6% (95% confidence interval (CI): 52.0–93.2; *P* < 0.001) (Fig. 2a). Of these 13 alive patients after 3 months of treatment initiation, six (46.2%) had type I LMD and seven (53.8%) had type II LMD, and the 3-month Kaplan–Meier OS rate was 66.7% (95% CI: 28.2–87.8) and 71.6% (95% CI: 35.0–89.9), respectively (Fig. 2b). Specifically, the 3-month Kaplan–Meier OS rate was 66.7% (95% CI: 33.7–86.0) in patients whose primary tumor location was the breast and 62.5% (95% CI: 14.2–89.3) in patients whose primary tumor location was the lung (Extended Data Fig. 1). Of note, the two patients who were not included in the ITT population were alive after 3 months of having started the treatment.

### Secondary outcome analysis

#### Efficacy endpoints

**Extended Data Fig. 2:**
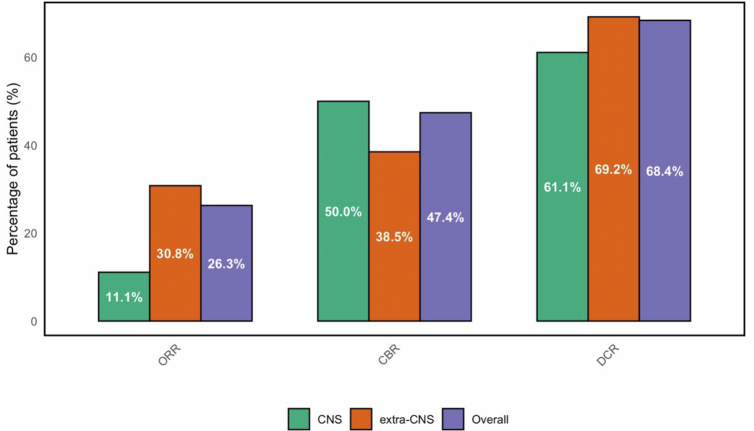
Percentage of patients with overall response rate (ORR), clinical benefit rate (CBR) and disease control rate (DCR) in patients with leptomeningeal disease from any solid tumor. Green bars represent central nervous system (CNS) lesions, orange bars represent extra-CNS lesions, and purple bars represent overall lesions.

**Extended Data Fig. 3:**
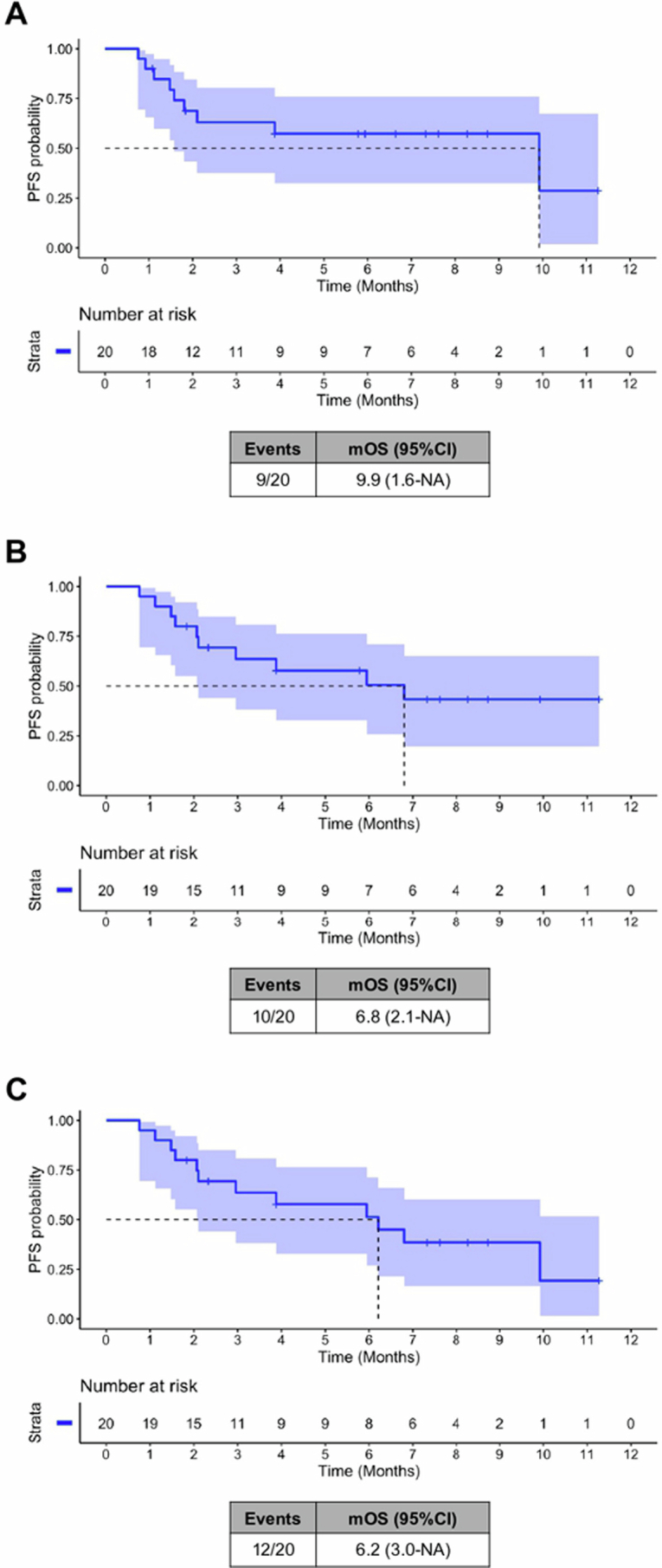
Kaplan Meier curves for progression-free survival of intracranial lesions as per RANO-BM criteria (A), and extracranial (B) and overall lesions (C) as per RECIST v1.1 criteria for patients with leptomeningeal disease from any solid tumor. Shaded areas represent 95% CI. CI: Confidence interval, mPFS: median progression-free survival, NA: Not achieved, PFS: Progression-free survival.

**Extended Data Fig. 4:**
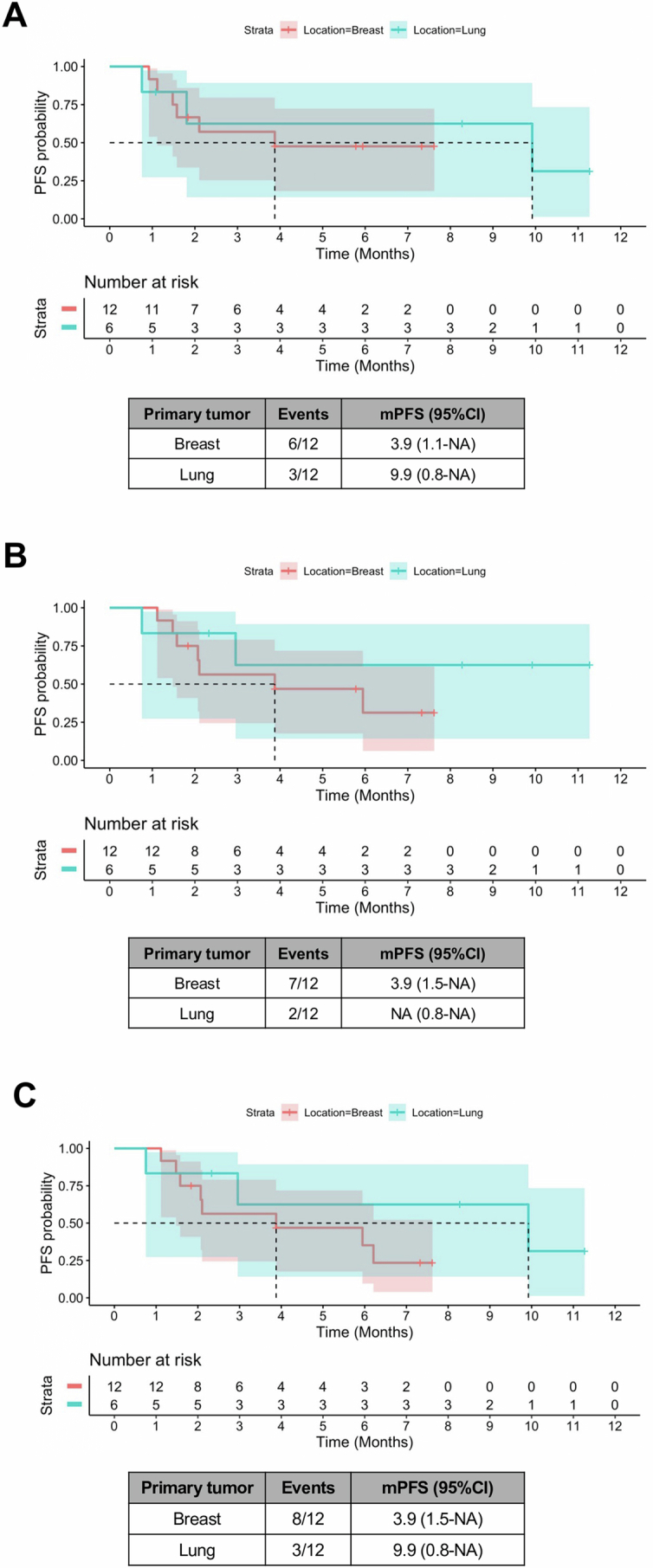
Kaplan Meier curves for progression-free survival of intracranial lesions as per RANO-BM criteria (A), and extracranial (B) and overall lesions (C) as per RECIST v1.1 criteria for patients with breast and lung cancers. Shaded areas represent 95% CI. CI: Confidence interval, mPFS: median progression-free survival, NA: Not achieved, PFS: Progression-free survival.

Investigator-assessed confirmed objective response rate (ORR) for intracranial lesions occurred in two of 18 (11.1% (95% CI: 1.4–34.7)) patients with parenchymal CNS lesions at baseline. Regarding extracranial and overall disease assessment, ORR was 30.8% (95% CI: 9.1–61.4; 4/13) and 26.3% (95% CI: 9.2–51.2; 5/19), respectively. None of the responder patients had received previous ADCs. Clinical benefit rate (CBR) for intracranial lesions was 50.0% (95% CI: 26.0–74.0; 9/18) and for extracranial and overall disease assessments was 38.5% (95% CI: 13.9–68.4; 5/13) and 47.4% (95% CI: 24.5–71.1; 9/19), respectively. Disease control rate (DCR) for intracranial lesions was 61.1% (95% CI: 35.8–82.7; 11/18) and for extracranial and overall disease assessments was 69.2% (95% CI: 38.6–90.9; 9/13) and 68.4% (95% CI: 43.5–87.4; 13/19), respectively (Extended Data Fig. 2). Median time to response (TTR) for intracranial disease was 1.8 months (95% CI: 1.6–1.9) and for extracranial and overall lesions was 2.0 months (95% CI: 1.9–2.1) and 2.0 months (95% CI: 1.6–2.1), respectively. Median duration of response (DoR) could not be calculated due to insufficient events. Median progression-free survival (PFS) for intracranial, extracranial and overall lesions was 9.9 months (95% CI: 1.6–not achieved (NA), 9/20), 6.8 months (95% CI: 2.1–NA, 10/20) and 6.2 months (95% CI: 3.0–NA, 12/20), with the upper bound of the CI not estimable due to insufficient events. Median OS was 10.5 months (95% CI: 2.1–NA), and the Kaplan–Meier-estimated 3-month and 6-month OS rates were 69.6 months (95% CI: 44.5–85.1) and 58.9 months (95% CI: 34.3–77.0), respectively. The Kaplan–Meier curves for PFS displaying the respective results for intracranial, extracranial and overall disease are shown in Extended Data Fig. 3 for all patients and in Extended Data Fig. 4 for those with breast and lung cancers. Efficacy endpoint results are summarized in Table 2.

**Table 2:** Secondary endpoints as per RANO-BM for intracranial lesions and as per RECIST version 1.1 for extracranial and overall lesions

Endpoint	RANO-BM (intracranial lesions; *n* = 18)*	RECIST version 1.1 (extracranial lesions; *n* = 13)*	RECIST version 1.1 (overall lesions; *n* = 19)*
ORR; % (95% CI); events	11.1 (1.4–34.7); 2/18	30.8 (9.1–61.4); 4/13	26.3 (9.2–51.2); 5/19
CBR; % (95% CI); events	50.0 (26.0–74.0); 9/18	38.5 (13.9–68.4); 5/13	47.4 (24.5–71.1); 9/19
DCR; % (95% CI); events	61.1 (35.8–82.7); 11/18	69.2 (38.6–90.9); 9/13	68.4 (43.5–87.4); 13/19
TTR; months (95% CI)	1.8 (1.6–1.9)	2.0 (1.9–2.1)	2.0 (1.7–2.1)
DoR; months (95% CI); events	NA (NA–NA); 0/1	4.8 (3.9–NA); 2/4	4.8 (4.6–NA); 3/5
PFS; months (95% CI); events	9.9 (1.6–NA); 9/20	6.8 (2.1–NA); 10/20	6.2 (3.0–NA); 12/20

*Two patients did not have intracranial lesions at baseline; seven patients did not have extracranial lesions at baseline; and one patient did not have any lesions at baseline. CBR, clinical benefit rate; CI, confidence interval; DCR, disease control rate; DoR, duration of response; NA, not achieved; ORR, overall response rate; PFS, progression-free survival; RANO-BM, response assessment in neuro-oncology for brain metastases; RECIST, response evaluation criteria in solid tumors; TTR, time to response.

Of note, according to the study protocol, CSF sampling was not mandatory, and its follow-up was conducted in only two patients. Therefore, no results regarding the evolution of cell counts over time are available.

#### Neurologic function and quality of life

**Extended Data Fig. 5:**
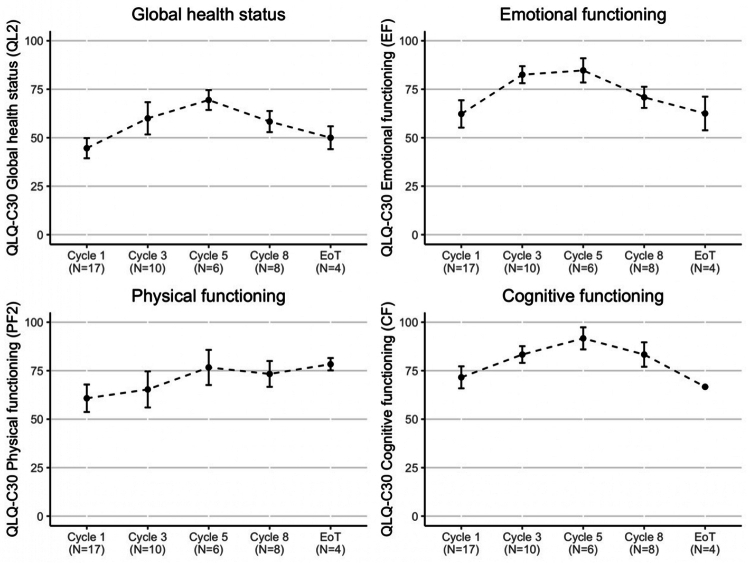
Parameters assessed using the EORTC QLQ-C30 questionnaire to evaluate the quality of life in patients with leptomeningeal disease from any solid tumor. The questionnaires were carried out during the treatment period in cycles 1, 3, 5 and 8, and end of treatment. N represents the number of patients assessed in each cycle.

**Extended Data Fig. 6:**
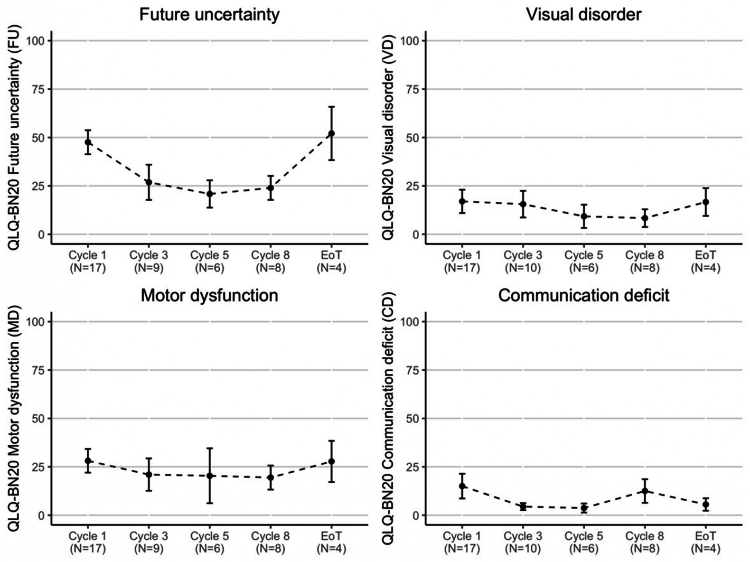
Parameters assessed using the EORTC QLQ-BN20 questionnaire to evaluate the quality of life in patients with leptomeningeal disease from any solid tumor. The questionnaires were carried out during the treatment period in cycles 1, 3, 5 and 8, and end of treatment. N represents the number of patients assessed in each cycle.

Twelve out of 20 (60%) patients had neurologic symptoms at baseline. At data cutoff, neurologic function evaluation as per Neurologic Assessment in Neuro-Oncology (NANO) scale reported two (10%) patients with neurologic response, 11 (55%) patients with neurologic stability and no patients with neurologic progression. The remaining seven (35%) patients could not be assessed due to the absence of post-baseline evaluations. QLQ-C30 and QLQ-BN20 questionaries were assessed to evaluate quality of life (QoL). Changes occurring during the follow-up using the QLQ-C30 questionnaire are shown in Extended Data Fig. 5 (for all patients) and in Supplementary Fig. 1 (per patient). Similarly, changes occurring during the follow-up using the QLQ-BN20 questionnaire are shown in Extended Data Fig. 6 (for all patients) and in Supplementary Fig. 2 (per patient).

#### Safety

TEAEs of any grade were observed in 21 (95.5%) patients (15 (68.2%) grade ≥3) (Tables 3 and 4). The most common TEAEs of any grade were the following: anemia (nine (40.9%) patients, one (4.5%) grade ≥3), neutropenia (six (27.3%) patients, three (13.6%) grade ≥3), thrombocytopenia (five (22.7%) patients, one (4.5%) grade ≥3), diarrhea (six (27.3%) patients, one (4.5%) grade ≥3), headache (five (22.7%) patients, one (4.5%) grade ≥3), nausea (seven (31.8%) patients, no grade ≥3) and asthenia (six (27.3%) patients, no grade ≥3) (Table 4). Treatment-related TEAEs were reported in 18 (81.8%) patients; the most common were nausea (six (27.3%) patients, no grade ≥3), anemia (seven (31.8%) patients, no grade ≥3), neutropenia (six (27.3%) patients, three (13.6%) grade ≥3) and thrombocytopenia (four (18.2%) patients, no grade ≥3) (Supplementary Table 2). Serious TEAEs were reported in 11 (50.0%) patients; the most common were headache and seizure (two (9.1%) patients, one (4.5%) grade ≥3) (Supplementary Table 3). Serious treatment-related TEAEs were reported in four (18.2%) patients, and three (13.6%) were grade ≥3 AEs, including dyspnea, interstitial lung disease (ILD) and febrile neutropenia (one (4.5%) patient each), all of which resolved (Supplementary Table 4). Respective patients with dyspnea and ILD discontinued treatment due to these TEAEs, whereas the patient with febrile neutropenia received no intervention; all patients fully recovered.

**Table 3:** Summary of AEs in patients with LMD from any solid tumor

Types of AEs, *n* (%)	*n* = 22
AEs	21 (95.5%)
TEAEs	21 (95.5%)
HER3-DXd-related TEAEs	18 (81.8%)
Serious TEAEs	11 (50.0%)
Serious HER3-DXd-related TEAEs	4 (18.2%)
Grade 3–5 TEAEs	15 (68.2%)
Grade 3–5 HER3-DXd-related TEAEs	7 (31.8%)
AESIs	1 (4.5%)
HER3-DXd-related AESIs	1 (4.5%)
Death due to TEAEs	0 (0.0%)
Death due to HER3-DXd-related TEAEs	0 (0.0%)
TEAEs leading to dose reduction	2 (9.1%)
TEAEs leading to dose interruption	6 (27.3%)
TEAEs leading to permanent discontinuation	1 (4.5%)

AEs: AEs, adverse events; AESIs, adverse events of special interest; HER3-DXd, patritumab deruxtecan; TEAEs, treatment-emergent adverse events.

**Table 4:** TEAEs in patients with LMD from any solid tumor

TEAEs, *n* (%)	Any grade	Grade ≥3
All	21 (95.5%)	15 (68.2%)
Blood and lymphatic system disorders	12 (54.6%)	5 (22.7%)
Anaemia	9 (40.9%)	1 (4.5%)
Neutropenia	6 (27.3%)	3 (13.6%)
Thrombocytopenia	5 (22.7%)	1 (4.5%)
Lymphopenia	3 (13.6%)	1 (4.5%)
Febrile neutropenia	1 (4.5%)	1 (4.5%)
General disorders and administration site conditions	12 (54.6%)	1 (4.5%)
Asthenia	6 (27.3%)	0 (0.0%)
Fatigue	1 (4.5%)	1 (4.5%)
Gastrointestinal disorders	13 (59.14%)	1 (4.5%)
Nausea	7 (31.8%)	0 (0.0%)
Diarrhea	6 (27.3%)	1 (4.5%)
Constipation	3 (13.6%)	0 (0.0%)
Vomiting	3 (13.6%)	0 (0.0%)
Ascites	1 (4.5%)	1 (4.5%)
Nervous system disorders	10 (45.5%)	3 (13.6%)
Headache	5 (22.7%)	1 (4.5%)
Dizziness	3 (13.6%)	0 (0.0%)
Disturbance in attention	2 (9.1%)	1 (4.5%)
Seizure	2 (9.1%)	1 (4.5%)
Investigations	7 (31.8%)	1 (4.5%)
Alanine aminotransferase increased	5 (22.7%)	0 (0.0%)
Aspartate aminotransferase increased	4 (18.2%)	0 (0.0%)
Transaminases increased	1 (4.5%)	1 (4.5%)
Metabolism and nutrition disorders	7 (31.8%)	1 (4.5%)
Hypocalcaemia	1 (4.5%)	1 (4.5%)
Skin and subcutaneous tissue disorders	7 (31.8%)	0 (0.0%)
Alopecia	3 (13.6%)	0 (0.0%)
Infections and infestations	7 (31.8%)	2 (9.1%)
Abdominal infection	1 (4.5%)	1 (4.5%)
Moraxella infection	1 (4.5%)	1 (4.5%)
Respiratory, thoracic and mediastinal disorders	6 (27.3%)	3 (13.6%)
ILD	2 (9.1%)	1 (4.5%)
Pulmonary embolism	2 (9.1%)	1 (4.5%)
Dyspnea	1 (4.5%)	1 (4.5%)
Musculoskeletal and connective tissue disorders	7 (31.8%)	2 (9.1%)
Muscular weakness	2 (9.1%)	2 (9.1%)
Fistula	1 (4.5%)	1 (4.5%)
Hepatobiliary disorders	1 (4.5%)	1 (4.5%)
Jaundice	1 (4.5%)	1 (4.5%)
Hyperbilirubinemia	1 (4.5%)	1 (4.5%)
Injury, poisoning and procedural complications	1 (4.5%)	1 (4.5%)
Fall	1 (4.5%)	1 (4.5%)
Neoplasms benign, malignant and unspecified (including cysts and polyps)	1 (4.5%)	1 (4.5%)
Cancer pain	1 (4.5%)	1 (4.5%)
ILD, interstitial lung disease; LMD, leptomeningeal disease; TEAEs, treatment-emergent adverse events.		

Dose interruption and permanent discontinuation of HER3-DXd because of TEAEs occurred in six (27.3%) patients and one (4.5%) patient, respectively. Dose reductions of HER3-DXd due to TEAEs occurred in two (9.1%) patients. There were no deaths due to HER3-DXd (Table 3).

### Pre-planned exploratory analysis

HER3 expression on baseline tumor tissue samples was examined in 15 available samples (two from the brain and 13 from extra-CNS sites) and assessed to evaluate whether there was correlation with efficacy endpoints (ORR, CBR, DCR, PFS and OS). The data did not reveal any correlation between the variables in any of the cases, as shown in Supplementary Fig. 3 and in Supplementary Table 5.

**Extended Data Fig. 7:**
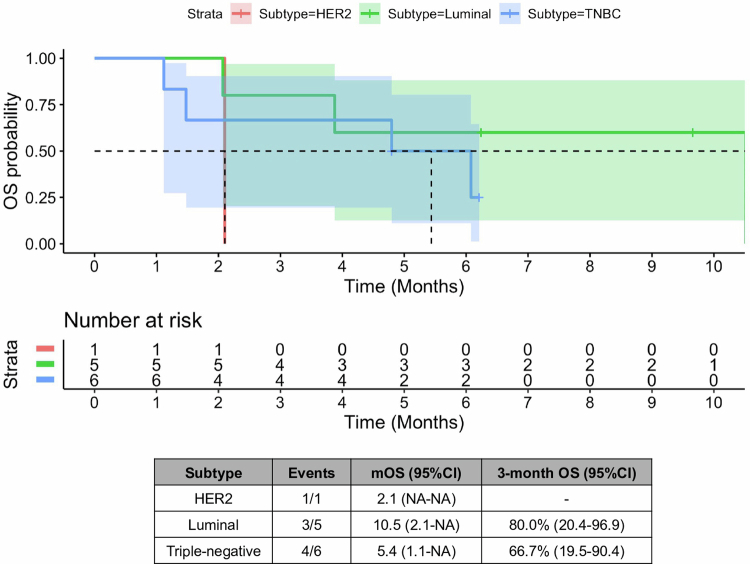
Kaplan Meier curves for overall survival in breast cancer patients with leptomeningeal disease by breast cancer subtype. Twelve out of the 20 participants in cohort 3 had breast cancer. In 3 of these 12 participants with breast cancer, the expression of hormonal receptors and HER2 could not be evaluated. Shaded areas represent 95% CI. CI: Confidence interval, HER2: Human epidermal growth factor receptor 2, mOS: median overall survival, NA: Not achieved, OS: Overall survival, TNBC: Triple-negative breast cancer.

Subgroup analyses for response and progression endpoints were not performed due to the small number of samples in each subgroup with the only exception of OS analysis, which was assessed by baseline breast cancer phenotype reported by sites (Extended Data Fig. 7).

## Discussion

To our knowledge, TUXEDO-3 is the first prospective clinical trial reporting activity of HER3-DXd in patients with LMD. The primary endpoint was met, with 65% of the ITT population alive beyond 3 months after enrollment. The predefined 25% threshold considered to indicate clinically relevant activity was clearly surpassed at a median follow-up time of 5.4 months. In addition, important clinical secondary endpoints, including symptom assessments and patient-reported outcomes, show the ability of the investigational agent to maintain or even improve patient well-being. Furthermore, radiological assessments document objective responses of parenchymal BMs, thus substantiating the evidence for the meaningful CNS efficacy of HER3-DXd. Overall, our data show clinically relevant activity of HER3-DXd in patients with LMD of solid cancers and may open the path to novel treatment options for this condition characterized by high morbidity and mortality.

Treatment options for patients with LMD are limited, and, in clinical practice, radiotherapy or intrathecal pharmacotherapy are oftentimes primarily considered. In this context, it is of interest to note that, although our trial allowed inclusion of patients with newly diagnosed LMD or LMD progressing after radiotherapy, only patients previously untreated for LMD were enrolled. Moreover, we observed a considerably higher accrual rate than initially projected, which allowed us to complete enrollment within 6 months. This enrollment pattern reflects the high unmet clinical need to identify new therapies for patients with LMD and supports the conduct of clinical trials with novel investigational agents in a first-line treatment setting.

Like previous trials, the inclusion criteria of our study allowed patients with LMD from any solid cancer in order to reflect the presentation of this condition across primary tumor entities as well as the evidence for HER3-DXd activity in various malignancies. In line with the epidemiology of LMD, most patients had breast or lung cancer, with only one patient each having melanoma and ovarian cancer as the primary tumor. Because confirmed responses to HER3-DXd have been seen across a wide range of baseline tumor HER3 membrane H-scores, clinical activity of HER3-DXd has so far not shown direct correlation with baseline tumoral HER3 expression levels^15,16,29^. In this sense, HER3-DXd has been demonstrated to enable a higher bystander effect than other previous ADCs, and a high expression of HER3 may not be necessary to make HER3-DXd more effective^29^. Because of this, together with the fact that no validated methods for assessment of HER3 in CSF samples are available, we enrolled patients irrespective of HER3 expression. This strategy is in line with that of other trials investigating HER3-DXd, such as HERTHENA-Breast01 (ref. ^19^), HERTHENA-Lung01 (ref. ^15^) or HERTHENA-Lung02 (ref. ^17^). However, the repeatedly suggested role of HER3 in CNS colonization provides a biological rationale for targeted treatment of secondary CNS involvement, particularly of breast and lung cancer. In addition, the analyses to find associations between HER3 expression and treatment response in this study were limited by the small sample size. Our results substantiate the role of HER3 as a relevant treatment target that should be further investigated in future studies.

Systemic treatments have increasingly been investigated in patients with LMD. Recent trials have reported promising outcomes with TKIs, such as osimertinib; immune checkpoint inhibitors, such as pembrolizumab, ipilimumab and nivolumab; and the ADC T-DXd^1^. However, differences in design, and particularly the choice of different primary endpoints, preclude meaningful comparison of the available clinical trial results. Given the difficulty of reliably assessing response to treatment in patients with LMD, we chose 3-month OS rate as the primary endpoint for our trial. This endpoint was used by two other recent clinical trials that met their predefined objectives and showed activity of systemically applied immune checkpoint inhibitors in this indication^29,30^. The 3-month OS rate of 65% and the Kaplan–Meier-estimated 3-month OS rate of 69.6% observed in our trial are higher than 60% achieved with pembrolizumab^29^ and 44% seen with the combination of ipilimumab and nivolumab^30^. However, differences of important baseline characteristics among the patients enrolled in those trials, including the distribution of primary tumor types, status of extracranial disease, clinical performance status and prior therapies, need to be acknowledged. For reliable definition of recommendations for the choice of specific systemic treatments for patients with LMD, prospective and adequately powered comparative studies will be necessary.

Although TUXEDO-3 is, to our knowledge, the first trial investigating HER3-DXd in patients with LMD, data in this disease setting are available for another ADC, as the activity of the related ADC T-DXd targeting HER2 in patients with LMD was investigated in a few small studies, and results corroborate evidence of T-DXd activity in LMD. The prospective multicohort phase 2 DEBBRAH trial enrolled a total of seven patients in cohort 5 specifically designed to assess T-DXd in patients with previously untreated pathologically confirmed LMD of HER2-positive and HER2-low breast cancer. In this small patient population, a median OS of 13.3 months, meeting the prespecified primary endpoint (median OS ≥6 months), was reported^27^. In the ROSET-BM study, 12-month PFS and OS were 60.7% and 87.1%, respectively, among 19 patients with LMD of HER2-positive breast cancer treated with T-DXd^31^. In a case series of eight patients with heavily pre-treated HER2-positive metastatic breast cancer and progressing LMD, all eight patients derived clinical benefit from T-DXd, and four (50%) patients had an objective partial response based on evaluations using the European Organization for Research and Treatment of Cancer (EORTC)/Response Assessment in Neuro-Oncology for Leptomeningeal Metastasis (RANO-LM) Revised Scorecard^32^. In another retrospective series of patients with LMD of various HER2-expressing tumor types, partial responses using the EORTC/RANO-LM Revised Scorecard were observed in six of 18 (33%) patients^33^. Taken together, the available data show that ADC as a substance class may have meaningful clinical activity in patients with LMD and should be further investigated in additional clinical trials. Further prospective and adequately powered studies are needed to compare the efficacy of T-DXd and HER3-DXd in patients with LMD and to inform future clinical practice guidelines.

Response assessment in patients with LMD is unreliable given the lack of clearly measurable disease manifestations in most patients^1^. LMD may present only as thin or diffuse contrast enhancement on magnetic resonance imaging (MRI) scans or with difficult-to-quantify amounts of tumor cells in CSF samples derived by lumbar punctures. As secondary endpoints, we included radiographic response assessment of parenchymal BMs and extracranial tumor manifestations in our trial. Using Response Assessment in Neuro-Oncology for Brain Metastases (RANO-BM) criteria, we documented objective and confirmed partial responses in two of 18 (11.1%) evaluable patients as well as a CBR of 50.0% and a DCR of 61.1%. These findings provide further support for the relevant CNS activity of HER3-DXd also noted in subgroup analyses of the HERTHENA-Lung01 trial^15^ and in cohorts 1 and 2 of TUXEDO-3, although higher intracranial response rates of 23–33% were documented in those studies. For extracranial tumors, we saw objective responses per Response Evaluation Criteria in Solid Tumors (RECIST) version 1.1 in four of 13 (30.8%) patients, which is in line with the extracranial response rates of up to 30% that have been reported for lung and breast cancer, respectively^15,16^. The median OS observed in our trial cohort of patients with LMD was 10.5 months and was 11.9 months in patients with advanced EGFR-mutated NSCLC without evidence of LMD enrolled in the HERTHENA-Lung01 trial^15^.

Regarding potential cross-resistance between topoisomerase I inhibitors, our results suggest no responses in patients pre-treated with T-DXd or sacituzumab govitecan (SG). None of the patients who showed radiological response based on the established criteria had received prior T-DXd, SG or other ADCs. In relation to the primary objective of this cohort, zero of one patient who had received prior T-DXd, two of five patients who had received prior SG and one of one patient who had received prior mirvetuximab were alive after 3 months of treatment initiation, suggesting potential variability in responses depending on prior treatment.

Concerning toxicity, no new safety signals were seen, and the safety profile of HER3-DXd in our patient cohort reflects that reported in other trials^15,16^. Notably, the rates of AEs affecting the nervous system, such as headache or dizziness, were not more common or severe than in trials using HER3-DXd in patients without CNS involvement. In addition to standard toxicity evaluations, we implemented structured assessments of neurological symptoms and QoL domains using standardized tools in our trial. We documented neurological stability or improvement in all evaluable patients as well as improvements in global health status, emotional functioning and cognitive functioning over the treatment period with HER3-DXd. These results are encouraging, as treatment recommendations for patients with LMD need to consider the palliative setting with high symptom burden and impaired QoL.

In conclusion, this study is, to our knowledge, the first prospective clinical trial investigating the novel HER3-targeting ADC HER3-DXd in patients with LMD. Cohort 3 of TUXEDO-3 met its primary endpoint and shows favorable results in a range of important secondary endpoints. Overall, our data indicate that HER3-DXd may be a useful treatment option with a manageable safety profile that may improve the prognosis and the symptomatic burden of patients with LMD. As limitations, the small sample size, the heterogeneous patient population, missing QoL data during follow-up and the relatively limited follow-up time on this cohort of patients need to be acknowledged because they limit subgroup analyses and may affect the interpretation of long-term outcomes, and further studies are warranted to define the optimal role of HER3-DXd in patients with LMD.

## Methods

TUXEDO-3 is an international, multicenter, single-arm, multicohort, phase 2 clinical trial evaluating the efficacy and safety of HER3-DXd in (1) patients with metastatic breast cancer with active BMs (that is, untreated or progressing after local treatment) (cohort 1); (2) patients with advanced NSCLC with active BMs (cohort 2); or (3) patients with treatment-naive LMD or LMD progressing after radiotherapy from any advanced solid tumor (cohort 3). In this paper, we report the results of cohort 3. The TUXEDO-3 study was conducted in accordance with the Declaration of Helsinki, the International Conference on Harmonization Good Clinical Practice guidelines and applicable regulations and laws from the recruiting countries (Austria and Spain). The study was approved by the ethics committee of the Instituto Valenciano de Oncología, Valencia (Spain). Written informed consent was obtained from each patient. None of patients received compensation for participation in the study. The trial is registered at ClinicalTrials.gov (NCT05865990) and the European Union Clinical Trials Register (EudraCT no. 2023-503251-10-00).

### Patients

Cohort 3 from the TUXEDO-3 trial consisted of patients with LMD from any advanced solid tumor. Key inclusion criteria were as follows: male or female patients ≥18 years of age with histologically documented solid tumor of any type and treatment-naive LMD or recurrence of LMD after radiotherapy; no need for immediate local treatment; either type I LMD, defined by positive CSF cytology or leptomeningeal biopsy, or type II LMD, defined by clinical findings and neuroimaging only, according to clinical practice guidelines jointly provided by the European Association of Neuro-Oncology (EANO) and the European Society for Medical Oncology (ESMO)^5^; life expectancy ≥6 weeks; left ventricular ejection fraction (LVEF) ≥ 50% as determined by multigated acquisition (MUGA) scan or echocardiogram; Karnofsky Performance Status (KPS) ≥ 70%; ECOG PS 0–2 (following the scale: 0 as fully active, 1 as light activity but limited with strenuous tasks, 2 as self-care but unable to work, 3 as staying in bed >50% of the day, 4 as completely disabled and 5 as deceased); and be able to understand the purpose of the study and give written informed consent. Key exclusion criteria were as follows: previous systemic therapy with any anti-HER3 directed drug; allergy or hypersensitivity to HER3-DXd or its components; treatment with approved or investigational cancer therapy within 14 days prior to initiation of study drug; LVEF < 50%; concurrent malignancy or malignancy within 5 years of study enrollment with the exception of carcinoma in situ of the cervix, non-melanoma skin carcinoma or stage I uterine cancer; CNS disorders; active cardiac disease or a history of cardiac dysfunction or conduction abnormalities within 6 months prior to the study; any serious medical condition or abnormality in clinical laboratory tests; current infection with hepatitis B virus, hepatitis C virus or HIV; major surgical procedure or significant traumatic injury within 21 days prior to randomization; and participants who are unable or unwilling to comply with the requirements of the protocol. A detailed list of all the inclusion and exclusion criteria can be found in Supplementary Table 6. In addition, given that these patients had LMD from any solid tumor, visceral disease was defined as metastatic involvement excluding organs with CNS lesions.

### Study procedures

In this study, HER3-DXd was administered intravenously at the standard dose of 5.6 mg kg^−1^ body weight on day 1 of each 21-day cycle until progression, unacceptable toxicity, death or withdrawal for any other reason. Before the first administration of the study drug, cranial MRI, bone scan and computed tomography (CT) scan of the chest and abdomen were carried out, if required to confirm LMD and according to local guidelines and clinical routine. Tumor staging investigations were conducted whenever disease progression was suspected. Cranial MRI and CT of the chest and abdomen were performed within 14 days of the next treatment cycle. At the investigator’s discretion, CT scans, MRI, CSF sampling and/or bone scans were obtained at any time when clinically indicated or if progressive disease was suspected. Imaging continued to be performed until radiologic evidence of disease progression, the start of new anti-cancer treatment, withdrawal of consent, death or the end of the study, whichever occurred first.

HER3, HER2, estrogen receptor (ER) and progesterone receptor (PgR) expressions were determined from baseline tissue samples during routine clinical diagnosis. Slides with 4-μm-thick, freshly cut tissue from formalin-fixed, paraffin-embedded (FFPE) tissue blocks were used. The expression of HER3 was determined in all patient samples using a proprietary assay developed by Ventana Medical Systems (Roche Tissue Diagnostics) and the primary antibody anti-HER3 SP438 clone (Roche Diagnostics) for immunohistochemical (IHC) staining. Slides from FFPE tissue blocks were stained with hematoxylin II and rabbit monoclonal negative control. The presence of HER3 was scored using the OptiView DAB IHC Detection Kit (Roche Tissue Diagnostics). The samples were scored for membrane percent positivity and intensity of 0 (no intensity), 1+ (weak intensity), 2+ (moderate intensity) or 3+ (strong intensity). HER3 expression was quantified using H-scores. The expression of HER2, ER and PgR was determined only in breast cancer patient samples using VENTANA anti-HER2/neu (4B5), CONFIRM anti-ER (SP1) and CONFIRM anti-PgR (1E2) clones (all Roche Diagnostics), respectively. Antibody dilutions were performed following the manufacturer’s instructions. An ultraView detection kit was used together with a BenchMark ULTRA system (Ventana Medical Systems), and visualization was performed using diaminobenzidine. All assessments were conducted by a pathologist. HER2-positive cases were classified following the 2023 American Society of Clinical Oncology (ASCO)–College of American Pathologists (CAP) guideline^34^. The samples included in this study were managed and processed by the biobank of the Ramón y Cajal Hospital-IRYCIS (Madrid, Spain, National Biobank Registry B.0000678), certified by ISO 9001:2015, following standardized procedures and using high-level databases of security.

### Endpoints

The primary endpoint of the TUXEDO-3 study for the third cohort of patients was the 3-month OS rate, defined as the rate of patients alive at 3 months after the start of the study treatment. Secondary endpoints included investigator-assessed ORR as per RANO-BM for intracranial lesions and as per RECIST version 1.1 for extracranial and overall lesions; investigator-assessed CBR, DCR, TTR, DoR and PFS as per RANO-BM for intracranial lesions and as per RECIST version 1.1 for extracranial and overall lesions; safety and toxicity of HER3-DXd according to National Cancer Institute Common Terminology Criteria for Adverse Events (NCI-CTCAE) version 5.0; QoL and neurocognitive function at cycles 1, 3, 5 and 8 and end of treatment using the EORTC QoL questionnaire (QLQ-C30) and the brain cancer–specific questionnaire (QLQ-BN20); and neurologic function at cycles 1, 3, 5 and 8 and end of treatment using the NANO scale of patients treated with HER3-DXd. Efficacy endpoints were analyzed according to HER3 expression levels in all patients. For those with breast cancer as the primary tumor, efficacy was also assessed based on ER, PgR and HER2 expression levels.

### Statistical analyses

TUXEDO-3 is a single-arm, non-comparative phase 2 trial that evaluated the safety and efficacy of HER3-DXd in BMs from metastatic breast cancer (cohort 1) and advanced NSCLC (cohort 2) and in LMD from any solid tumor (cohort 3). For the third cohort, the primary endpoint was the number of patients alive after 3 months of treatment initiation (3-month OS) in the ITT population. We planned to assign 20 patients to this cohort. The protocol specified one interim analysis with 10 evaluable patients, based on Simon’s two-stage design. The study would continue with the second stage if one or more patients alive after 3 months was observed. The critical value for the final analysis in this cohort was three or more patients out of 20 alive after 3 months. The null hypothesis could have been rejected if 15% or more of patients were alive at 3 months. This design yielded a type I error rate of 10% and a power of 88% to reject the null hypothesis. *P* values and 95% CIs using the Koyama and Chen method^35^ were estimated. Efficacy endpoints were assessed in all patients who received at least one dose of the study drug and met the inclusion criteria. ORR, CBR and DCR were estimated with 95% Clopper–Pearson CI. CBR was defined as the rate of patients with objective response, including confirmed complete or partial responses or stable disease (SD) for at least 24 weeks. DCR was defined as the rate of patients with objective response or SD. DoR and TTR were analyzed in all patients who had an objective response and were summarized with median and with 95% CI. PFS, OS and the estimated 3-month OS rate were estimated using the Kaplan–Meier method, reporting the number of events and the median with 95% CI. Results from QoL questionnaires were summarized with median and 95% CI. Descriptive statistics were used for summarizing safety data. The association between biomarker expression and HER3-DXd activity was assessed using the Wilcoxon rank-sum test (Mann–Whitney *U*-test) for binary variables and Cox regression for time-to-event variables. HER3 expression was analyzed as a continuous variable because there was not a validated cutoff to categorize HER3 expression into groups (for example, expression versus no expression and low expression versus high expression). The Wald test was used for hypothesis testing. Two-sided *P* values with alpha ≤ 0.05 level of significance were used for all analyses, except for those involving HER3 expression due to the exploratory nature of this analysis. Data analysis was performed using R software (version 4.3.2) within the RStudio environment (version 2023.12.2 + 402).

### Reporting summary

Further information on research design is available in the Nature Portfolio Reporting Summary linked to this article.

## Online content

Any methods, additional references, Nature Portfolio reporting summaries, source data, extended data, supplementary information, acknowledgements, peer review information; details of author contributions and competing interests; and statements of data and code availability are available at 10.1038/s41591-025-03744-1.

## Supplementary information

Supplementary Information Supplementary Figs. 1–3 and Supplementary Tables 1–6.

Reporting Summary

## Data Availability

Data collected within the TUXEDO-3 study will be made available to researchers upon reasonable request. Access to the data is controlled to ensure the protection of participant privacy and compliance with applicable data protection laws and regulations. Data will be shared upon revision and approval based on scientific merit by the TUXEDO-3 management group (which includes a qualified statistician) of a detailed proposal for their use. The data required for the approved, specified purposes and the trial protocol will be provided after the completion of a data-sharing agreement that will be set up by the study sponsor (MEDSIR). All data provided will be anonymized to respect the privacy of patients who have participated in the trial. Estimated timeframe for response will be within 30 days. Requests for data should be addressed to the corresponding author (M.P.; matthias.preusser@meduniwien.ac.at).

## References

[1] Ozair, A. et al. Leptomeningeal metastatic disease: new frontiers and future directions. *Nat. Rev. Clin. Oncol.* **22**, 134–154 (2025). 39653782 10.1038/s41571-024-00970-3

[2] Wilcox, J. A. et al. Leptomeningeal metastases from solid tumors: a Society for Neuro-Oncology and American Society of Clinical Oncology consensus review on clinical management and future directions. *Neuro Oncol.* **26**, 1781–1804 (2024). 38902944 10.1093/neuonc/noae103 PMC11449070

[3] Le Rhun, E. et al. Prolonged survival of patients with breast cancer-related leptomeningeal metastases. *Anticancer Res.* **33**, 2057–2063 (2013). 23645756

[4] Bartsch, R., Jerzak, K. J., Larrouquere, L., Müller, V. & Le Rhun, E. Pharmacotherapy for leptomeningeal disease in breast cancer. *Cancer Treat. Rev.* **122**, 102653 (2024). 38118373 10.1016/j.ctrv.2023.102653

[5] Le Rhun, E. et al. Leptomeningeal metastasis from solid tumours: EANO–ESMO Clinical Practice Guideline for diagnosis, treatment and follow-up. *ESMO Open* **8**, 101624 (2023). 37863528 10.1016/j.esmoop.2023.101624 PMC10619142

[6] Gao, L. et al. HER3: updates and current biology function, targeted therapy and pathologic detecting methods. *Life Sci.* **357**, 123087 (2024). 39366553 10.1016/j.lfs.2024.123087

[7] Uliano, J., Corvaja, C., Curigliano, G. & Tarantino, P. Targeting HER3 for cancer treatment: a new horizon for an old target. *ESMO Open* **8**, 100790 (2023). 36764093 10.1016/j.esmoop.2023.100790 PMC9929675

[8] Li, Q. et al. Prognostic significance of HER3 in patients with malignant solid tumors. *Oncotarget* **8**, 67140–67151 (2017). 28978022 10.18632/oncotarget.18007 PMC5620162

[9] Gandullo-Sánchez, L., Ocaña, A. & Pandiella, A. HER3 in cancer: from the bench to the bedside. *J. Exp. Clin. Cancer Res.* **41**, 310 (2022). 36271429 10.1186/s13046-022-02515-x PMC9585794

[10] Da Silva, L. et al. HER3 and downstream pathways are involved in colonization of brain metastases from breast cancer. *Breast Cancer Res.* **12**, R46 (2010). 20604919 10.1186/bcr2603 PMC2949633

[11] Saunus, J. M. et al. Integrated genomic and transcriptomic analysis of human brain metastases identifies alterations of potential clinical significance. *J. Pathol.* **237**, 363–378 (2015). 26172396 10.1002/path.4583

[12] Sun, M. et al. HER family receptor abnormalities in lung cancer brain metastases and corresponding primary tumors. *Clin. Cancer Res.* **15**, 4829–4837 (2009). 19622585 10.1158/1078-0432.CCR-08-2921 PMC3372920

[13] Lim, M. et al. Innovative therapeutic strategies for effective treatment of brain metastases. *Int. J. Mol. Sci.* **20**, 1280 (2019). 30875730 10.3390/ijms20061280 PMC6471202

[14] Tomasich, E. et al. Frequent overexpression of HER3 in brain metastases from breast and lung cancer. *Clin. Cancer Res.* **29**, 3225–3236 (2023). 37036472 10.1158/1078-0432.CCR-23-0020

[15] Yu, H. A. et al. HERTHENA-Lung01, a phase II trial of patritumab deruxtecan (HER3-DXd) in epidermal growth factor receptor–mutated non–small-cell lung cancer after epidermal growth factor receptor tyrosine kinase inhibitor therapy and platinum-based chemotherapy. *J. Clin. Oncol.* **41**, 5363–5375 (2023). 37689979 10.1200/JCO.23.01476 PMC10713116

[16] Krop, I. E. et al. Patritumab deruxtecan (HER3-DXd), a human epidermal growth factor receptor 3–directed antibody-drug conjugate, in patients with previously treated human epidermal growth factor receptor 3–expressing metastatic breast cancer: a multicenter, phase I/II trial. *J. Clin. Oncol.* **41**, 5550–5560 (2023). 37801674 10.1200/JCO.23.00882 PMC10730028

[17] Mok, T. et al. HERTHENA-Lung02: phase III study of patritumab deruxtecan in advanced *EGFR*-mutated NSCLC after a third-generation EGFR TKI. *Future Oncol.* **20**, 969–980 (2024). 38095056 10.2217/fon-2023-0602

[18] Powles, T. B. et al. HERTHENA-PanTumor01: a global phase II trial of HER3-DXd in metastatic solid tumors. *Ann. Oncol.* **35**, S534–S535 (2024).

[19] Merck Sharp & Dohme LLC. Study of patritumab deruxtecan with other anticancer agents in participants with HER2 positive breast cancer that has spread and cannot be surgically removed (MK-1022-009). https://www.clinicaltrials.gov/study/NCT06686394

[20] The Netherlands Cancer Institute. Whole body HER3 quantification with radiolabelled patritumab deruxtecan (HER3-DXd) PET/CT. https://www.clinicaltrials.gov/study/NCT06222489

[21] Mair, M. J. et al. Understanding the activity of antibody–drug conjugates in primary and secondary brain tumours. *Nat. Rev. Clin. Oncol.* **20**, 372–389 (2023). 37085569 10.1038/s41571-023-00756-z

[22] Bartsch, R. et al. Trastuzumab deruxtecan in HER2-positive breast cancer with brain metastases: a single-arm, phase 2 trial. *Nat. Med.* **28**, 1840–1847 (2022). 35941372 10.1038/s41591-022-01935-8 PMC9499862

[23] Bartsch, R. et al. Results of a patient-level pooled analysis of three studies of trastuzumab deruxtecan in HER2-positive breast cancer with active brain metastasis. *ESMO Open* **10**, 104092 (2025). 39754978 10.1016/j.esmoop.2024.104092 PMC11758132

[24] Bartsch, R. et al. Final outcome analysis from the phase II TUXEDO-1 trial of trastuzumab-deruxtecan in HER2-positive breast cancer patients with active brain metastases. *Neuro Oncol.* **26**, 2305–2315 (2024). 38963808 10.1093/neuonc/noae123 PMC11630562

[25] Harbeck, N. et al. Publisher Correction: Trastuzumab deruxtecan in HER2-positive advanced breast cancer with or without brain metastases: a phase 3b/4 trial. *Nat. Med.* **30**, 3780 (2024). 39653780 10.1038/s41591-024-03349-0 PMC11645286

[26] Gennari, A. et al. ESMO Clinical Practice Guideline for the diagnosis, staging and treatment of patients with metastatic breast cancer. *Ann. Oncol.* **32**, 1475–1495 (2021). 34678411 10.1016/j.annonc.2021.09.019

[27] Vaz Batista, M. et al. The DEBBRAH trial: trastuzumab deruxtecan in HER2-positive and HER2-low breast cancer patients with leptomeningeal carcinomatosis. *Med* **6**, 100502 (2025). 10.1016/j.medj.2024.08.001 39265579

[28] Nakayama, T. et al. Trastuzumab deruxtecan for the treatment of patients with HER2-positive breast cancer with brain and/or leptomeningeal metastases: an updated overall survival analysis using data from a multicenter retrospective study (ROSET-BM). *Breast Cancer* **31**, 1167–1175 (2024). 39133378 10.1007/s12282-024-01614-1 PMC11489233

[29] Brastianos, P. K. et al. Single-arm, open-label phase 2 trial of pembrolizumab in patients with leptomeningeal carcinomatosis. *Nat. Med.* **26**, 1280–1284 (2020). 32483359 10.1038/s41591-020-0918-0

[30] Brastianos, P. K. et al. Phase II study of ipilimumab and nivolumab in leptomeningeal carcinomatosis. *Nat. Commun.* **12**, 5954 (2021). 34642329 10.1038/s41467-021-25859-y PMC8511104

[31] Niikura, N. et al. Treatment with trastuzumab deruxtecan in patients with HER2-positive breast cancer and brain metastases and/or leptomeningeal disease (ROSET-BM). *NPJ Breast Cancer* **9**, 82 (2023). 37821514 10.1038/s41523-023-00584-5 PMC10567705

[32] Alder, L. et al. Durable responses in patients with HER2+ breast cancer and leptomeningeal metastases treated with trastuzumab deruxtecan. *NPJ Breast Cancer* **9**, 19 (2023). 36997605 10.1038/s41523-023-00519-0 PMC10063529

[33] Rogawski, D. et al. Durable responses to trastuzumab deruxtecan in patients with leptomeningeal metastases from breast cancer with variable HER2 expression. *J. Neurooncol.* **170**, 209–217 (2024). 39073687 10.1007/s11060-024-04788-y

[34] Wolff, A. C. et al. Human epidermal growth factor receptor 2 testing in breast cancer: ASCO–College of American Pathologists Guideline Update. *J. Clin. Oncol.* **41**, 3867–3872 (2023). 37284804 10.1200/JCO.22.02864

[35] Koyama, T. & Chen, H. Proper inference from Simon’s two-stage designs. *Stat. Med.* **27**, 3145–3154 (2008). 17960777 10.1002/sim.3123 PMC6047527

