# Supplementary material for: Patritumab deruxtecan in leptomeningeal metastatic disease of solid tumors: the phase 2 TUXEDO-3 trial
Source: Nat Med. 2025 May 30;31(8):2797–805. doi: 10.1038/s41591-025-03744-1 (PMC12353872; doi:10.1038/s41591-025-03744-1)
Supplement: Supplementary file 1 — Supplementary Figs. 1–3 and Supplementary Tables 1–6. [file 41591_2025_3744_MOESM1_ESM.pdf]

nature medicine

Supplementary information https://doi.org/10.1038/s41591-025-03744-1

Patritumab deruxtecan in leptomeningeal metastatic disease of solid tumors: the phase 2 TUXEDO-3 trial

In the format provided by the authors and unedited

Patritumab deruxtecan in leptomeningeal metastatic disease of solid tumors: the phase 2 TUXEDO-3 trial

Supplementary material

Supplementary Figures. 2

Supplementary Fig. 1. Parameters assessed using the EORTC QLQ-C30 questionnaire per patient. 2

Supplementary Fig. 2. Parameters assessed using the EORTC QLQ-BN20 questionnaire per patient. 2

Supplementary Fig. 3. Box plots comparing HER3 expression with overall response rate (ORR), clinical benefit rate (CBR), and disease control rate (DCR) for intracranial lesions as per RANO-BM criteria (A), and extracranial (B) and overall lesions (C) as per RECIST v1.1 criteria for patients with leptomeningeal disease from any solid tumor. 3

Supplementary Tables 3

Supplementary Table 1. Protocol deviations of the TUXEDO3 clinical trial within the cohort of LMD patients from any solid tumor. 3

Supplementary Table 2. HER3-DXd-related treatment-emergent adverse events (TEAEs) in patients with leptomeningeal disease from any solid tumor. 4

Supplementary Table 3. Serious treatment-emergent adverse events (TEAEs) in patients with leptomeningeal disease from any solid tumor. 4

Supplementary Table 4. Serious HER3-DXd-related treatment-emergent adverse events (TEAEs) in patients with leptomeningeal disease from any solid tumor. 5

Supplementary Table 5. Correlation between HER3 expression and progression-free survival (PFS) and overall survival (OS) for patients with leptomeningeal disease from any solid tumor in the TUXEDO-3 study. 5

Supplementary Table 6. Eligibility criteria for patients with leptomeningeal disease (LMD) from any advanced solid tumor from the TUXEDO-3 study. 5

Supplementary Figures

Supplementary Fig. 1. Parameters assessed using the EORTC QLQ-C30 questionnaire per patient. Each color represents one different patient.

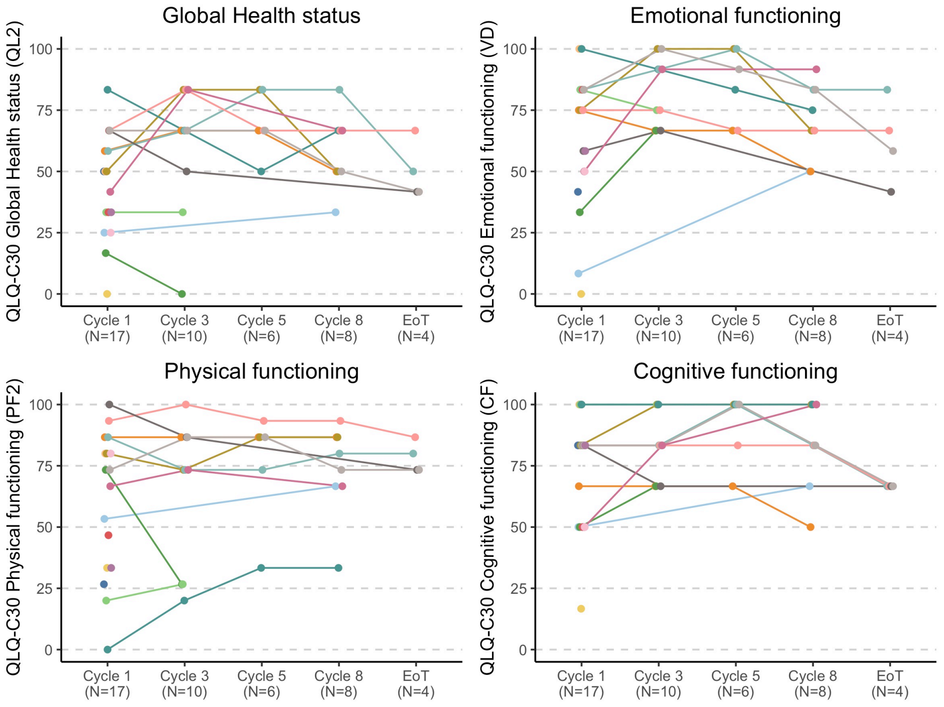

Supplementary Fig. 2. Parameters assessed using the EORTC QLQ-BN20 questionnaire per patient. Each color represents one different patient.

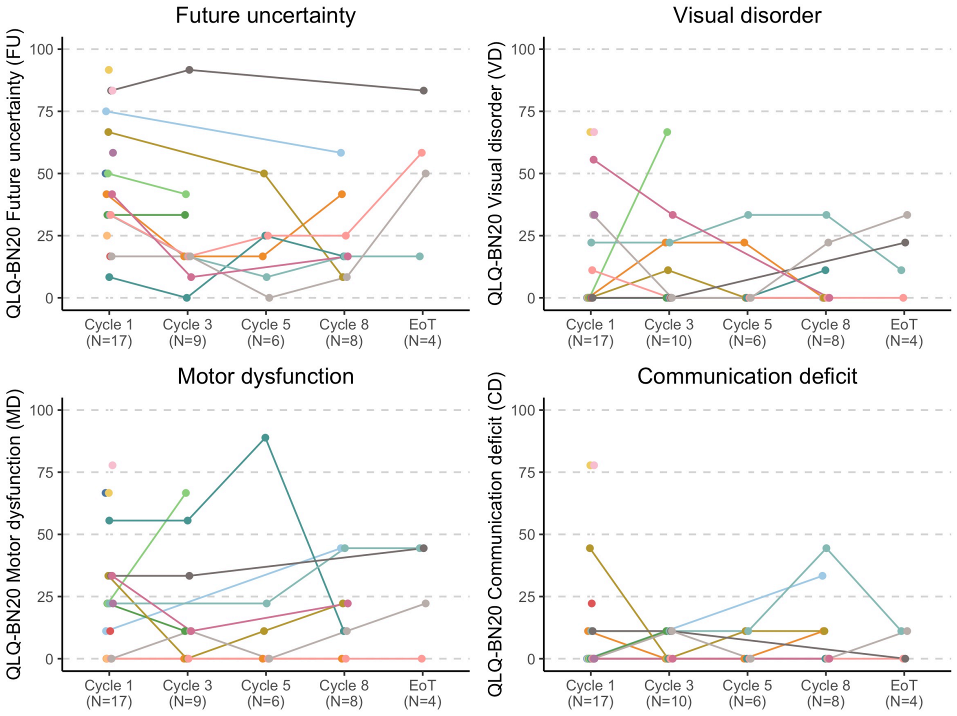

Supplementary Fig. 3. Box plots comparing HER3 expression with overall response rate (ORR), clinical benefit rate (CBR), and disease control rate (DCR) for intracranial lesions as per RANO-BM criteria **(A)**, and extracranial **(B)** and overall lesions **(C)** as per RECIST v1.1 criteria for patients with leptomeningeal disease from any solid tumor. Yes: clinical response; no: no response.

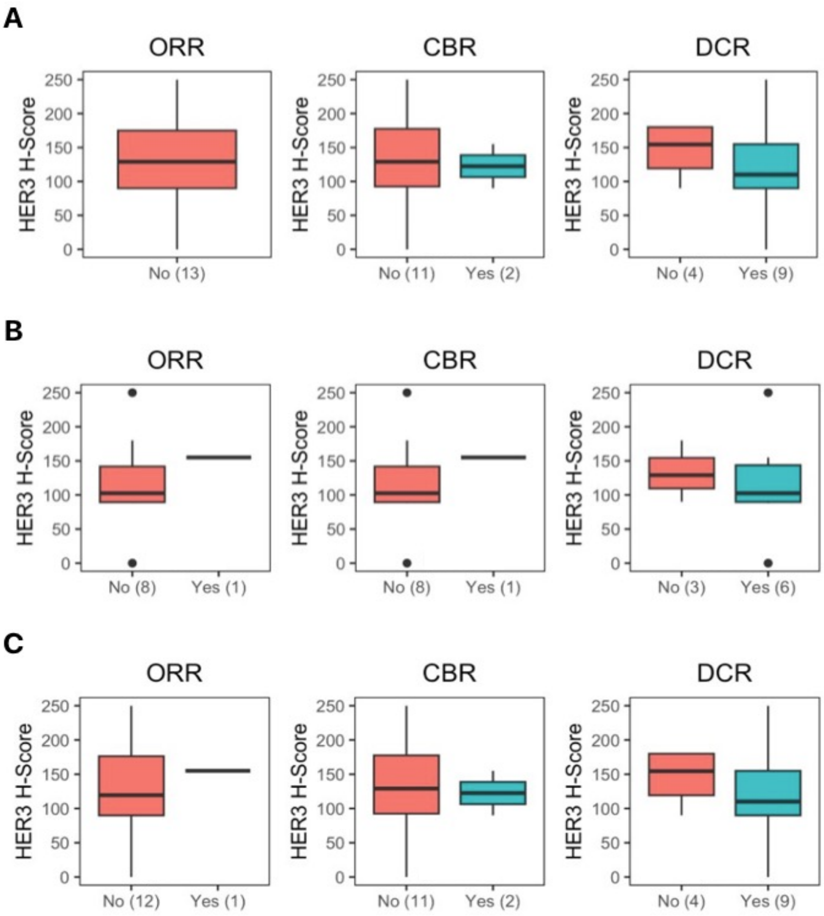

Supplementary Tables

Supplementary Table 1. Protocol deviations of the TUXEDO3 clinical trial within the cohort of LMD patients from any solid tumor.

Patient	Description	Action Taken
**107-001**	Patient did not fulfil specific criteria for cohort 3 from the TUXEDO-3 study since the patient had received previous systemic treatment for LMD before the enrolment.	Excluded from the intention-to-treat (ITT) population but included in the safety population analysis.
**101-003**	Patient did not fulfil specific criteria for cohort 3 from the TUXEDO-3 study since the patient had received previous systemic treatment for LMD before enrolment.	Excluded from the ITT population but included in the safety population analysis.

**101-020**	Undertreated (4.2 mg/kg vs. 4.8 mg/kg) during two cycles because the pharmacy program was not updated.	Included in both the ITT and safety populations analyses.

Supplementary Table 2. HER3-DXd-related treatment-emergent adverse events (TEAEs) in patients with leptomeningeal disease from any solid tumor.

HER3-DXd-related TEAEs, n (%)	Any Grade	Grade ≥3
**All**	**18 (81.8%)**	**7 (31.8%)**
**Gastrointestinal disorders**	**10 (45.5%)**	**0 (0.0%)**
Nausea	6 (27.3%)	0 (0.0%)
Diarrhoea	3 (13.6%)	0 (0.0%)
**Blood and lymphatic system disorders**	**10 (45.5%)**	**4 (18.2%)**
Anaemia	7 (31.8%)	0 (0.0%)
Neutropenia	6 (27.3%)	3 (13.6%)
Thrombocytopenia	4 (18.2%)	0 (0.0%)
Lymphopenia	2 (9.1%)	1 (4.5%)
Febrile neutropenia	1 (4.5%)	1 (4.5%)
**Investigations**	**6 (27.3%)**	**1 (4.5%)**
Alanine aminotransferase increased	3 (13.6%)	0 (0.0%)
Aspartate aminotransferase increased		0 (0.0%)
Transaminases increased	3 (13.6%)	1 (4.5%)
	1 (4.5%)	
**Skin and subcutaneous tissue disorders**	**5 (22.7%)**	**0 (0.0%)**
Alopecia	3 (13.6%)	0 (0.0%)
**Respiratory, thoracic and mediastinal**	**3 (13.6%)**	**2 (9.1%)**
**disorders**	2 (9.1%)	1 (4.5%)
Interstitial lung disease	1 (4.5%)	1 (4.5%)
Dyspnea		
**General disorders and administration site conditions**	**3 (13.6%)**	**0 (0.0%)**
Asthenia	3 (13.6%)	0 (0.0%)

Supplementary Table 3. Serious treatment-emergent adverse events (TEAEs) in patients with leptomeningeal disease from any solid tumor.

Serious TEAEs, n (%)	Any Grade	Grade ≥3
**All**	**11 (50.0%)**	**9 (40.9%)**
**Gastrointestinal disorders**	**4 (18.2%)**	**1 (4.5%)**
Ascites	1 (4.5%)	1 (4.5%)
**Respiratory, thoracic and mediastinal**	**3 (13.6%)**	**3 (13.6%)**
**disorders**	1 (4.5%)	1 (4.5%)
Dyspnea	1 (4.5%)	1 (4.5%)
Interstitial lung disease	1 (4.5%)	1 (4.5%)
Pulmonary embolism		
**Nervous system disorders**	**4 (18.2%)**	**3 (13.6%)**
Headache	2 (9.1%)	1 (4.5%)
Seizure	2 (9.1%)	1 (4.5%)
Disturbance in attention	1 (4.5%)	1 (4.5%)

**Blood and lymphatic system disorders**	**2 (9.1%)**	**1 (4.5%)**
Febrile neutropenia	1 (4.5%)	1 (4.5%)
**Hepatobiliary disorders**	**1 (4.5%)**	**1 (4.5%)**
Jaundice	1 (4.5%)	1 (4.5%)
**Infections and infestations**	**2 (9.1%)**	**2 (9.1%)**
Abdominal infection	1 (4.5%)	1 (4.5%)
Moraxella infection	1 (4.5%)	1 (4.5%)
**Neoplasms benign, malignant and unspecified (including cysts and polyps)**	**1 (4.5%)**	**1 (4.5%)**
Cancer pain	1 (4.5%)	1 (4.5%)

Supplementary Table 4. Serious HER3-DXd-related treatment-emergent adverse events (TEAEs) in patients with leptomeningeal disease from any solid tumor.

Serious HER3-DXd-related TEAEs, n (%)	Any Grade	Grade ≥3
**All**	**4 (18.2%)**	**3 (13.6%)**
**Respiratory, thoracic and mediastinal disorders**	**2 (9.1%)**	**2 (9.1%)**
Dyspnea	1 (4.5%)	1 (4.5%)
Interstitial lung disease	1 (4.5%)	1 (4.5%)
**Blood and lymphatic system disorders**	**1 (4.5%)**	**1 (4.5%)**
Febrile neutropenia	1 (4.5%)	1 (4.5%)

Supplementary Table 5. Correlation between HER3 expression and progression-free survival (PFS) and overall survival (OS) for patients with leptomeningeal disease from any solid tumor in the TUXEDO-3 study. CI: Confidence interval, HR: Hazard ratio.

	**HER3 expression**		
**Efficacy endpoint**	**Events**	**HR (95% CI)**	**P-value**
PFS (RANO-BM for intracranial lesions)	4/15	1.0 (0.99; 1.02)	0.6009
PFS (RECIST v.1.1 for extracranial lesions)	4/15	1.0 (0.99; 1.02)	0.5608
PFS (RECIST v.1.1 for overall lesions)	4/15	1.0 (0.99; 1.02)	0.5344
OS	5/15	1.0 (0.99; 1.03)	0.2599

Supplementary Table 6. Eligibility criteria for patients with leptomeningeal disease (LMD) from any advanced solid tumor from the TUXEDO-3 study.

Inclusion criteria

To be eligible for inclusion in the cohort 3 of the TUXEDO-3 study, each patient had to meet all of the following inclusion criteria:

1. Patient must be capable of understanding the purpose of the study and have signed written informed consent form (ICF) prior to beginning specific protocol procedures.
2. Age ≥ 18 years at the time of signing ICF.

3. Histologically documented solid tumor of any type.
4. Type I LMD, defined by positive CSF cytology or leptomeningeal biopsy, or type II LMD, defined by clinical findings and neuroimaging only, according to European Society for Molecular Oncology Standard Operating Procedures for Clinical Practice Guideline 2017.
5. Newly diagnosed LMD or LMD progressing after radiotherapy.
6. Life expectancy ≥ 6 weeks.
7. Karnofsky Performance Status (KPS) ≥70%, Eastern Cooperative Oncology Group (ECOG) performance status (PS) ≤ 2.
8. Patients must be able to tolerate therapy.
9. Availability and willingness to provide the most recently available tumor tissue sample (formalin-fixed and paraffin-embedded [FFPE], no cytology/cell block, no bone/decalcified bone sample) of primary tumor or any metastatic site from biopsy collected after last round of prior treatment and ≤ 6 months prior to HER3-DXd, if possible, at the time of inclusion for retrospective exploratory biomarker testing. If archival tissue is not available, a newly obtained baseline biopsy of an accessible tumor lesion is required prior to start of study treatment (unless not possible because of inaccessible tumor location or safety concerns). Collection and/or shipment of pre-treatment tumor tissue biopsy for retrospective biomarker testing should be at least initiated treatment at the time of inclusion.
10. No indication for immediate local therapy (neurosurgery, brain radiotherapy).
11. Patient has adequate bone marrow, liver, and renal function:
    a. Hematological (without platelet, red blood cell transfusion, and/or granulocyte colony-stimulating factor support within 14 days prior to hematological assessments during the screening period): White blood cell (WBC) count > 3.0 x 10^9/L, absolute neutrophil count (ANC) ≥ 1.5 x 10^9/L, platelet count ≥ 100.0 x10^9/L, and hemoglobin ≥ 10.0 g/dL (≥ 6.2 mmol/L).
    b. Hepatic: Serum albumin ≥ 2.5 g/dL; total bilirubin ≤ 1.5 times upper limit of normal (ULN) (≤ 3 in patients with liver metastases or known history of Gilbert’s disease); both alkaline phosphatase (ALP) and Gamma Glutamyl Transferase (GGT) ≤ 2.5 times ULN (ALP ≤ 5 times ULN in patients with liver and/or bone metastases, and GGT increased in patients with liver metastases); aspartate transaminase (AST); alanine transaminase (ALT) ≤ 3 times ULN (≤ 5 times ULN in patients with liver metastases); international

normalized ratio (INR) < 1.5. Prothrombin time (PT) or Prothrombin time-international normalized ratio (PT-INR) and activated partial thromboplastin time (aPTT)/partial thromboplastin time (PTT) ≤ 1.5 × ULN, except for subjects receiving coumarin-derivative anticoagulants, factor Xa inhibitors, or other similar anticoagulant therapy, who must have PT-INR within therapeutic range as deemed appropriate by the Investigator from product safety requirements (PSR).

c. Renal: serum creatinine ≤ 1.5 x ULN or creatinine clearance ≥ 50 mL/min/1.73 m2 based on Cockcroft−Gault glomerular filtration rate estimation for patients with creatinine levels above institutional normal.

12. Resolution of all acute toxic effects of prior anti-cancer therapy to grade ≤ 1 as determined by the US National Cancer Institute (NCI)-Common Terminology Criteria for Adverse Events (CTCAE) version 5.0 (v.5.0).

    Note: Except for alopecia or other toxicities not considered a safety risk for the patient at investigator's discretion.

13. For women of childbearing potential: agreement to remain abstinent (must refrain from heterosexual intercourse) or use highly effective contraceptive methods, or two effective contraceptive methods, as defined in the CSP, during the treatment period and for at least 7 months after the last dose of study treatment, whichever is longer. Women of childbearing potential must have a negative serum pregnancy test within 14 days before study treatment initiation (with result available prior to dosing) and must agree to refrain from donating eggs during the entire study treatment period and for 7 months after the last administration of the study drug.
14. For male subjects: being surgically sterile or having agreed to true abstinence (must refrain from heterosexual intercourse) or having female partners willing to agree with true abstinence or use barrier contraceptive measures mentioned above during the entire study treatment period and for 4 months after the last administration of the study drug. Male patients must agree to refrain from donating sperm during the entire study treatment period and for 4 months after the last administration of the study drug.
15. Patients must be accessible for treatment and follow-up.

**Exclusion criteria**
Patients were excluded from the study if they meet any of the following criteria:

1. Current participation in another therapeutic clinical trial.
2. Treatment with approved or investigational cancer therapy within 14 days prior to initiation of study drug.
3. Patients have a concurrent malignancy or malignancy within five years of study enrollment except for carcinoma in situ of the cervix, non-melanoma skin carcinoma, or stage I uterine cancer. For other cancers considered to have a low risk of recurrence, discussion with the Medical Monitor is required.
4. Previous systemic therapy with any anti-HER3 directed drug.
5. Known allergy or hypersensitivity to HER3-DXd or any of the drug components.
6. Radiotherapy or limited-field palliative radiotherapy within seven days prior to study enrolment, or patients who have not recovered from radiotherapy-related toxicities to baseline or grade ≤ 1 and/or from whom ≥ 25% of the bone marrow has been previously irradiated.
7. Patients with an active cardiac disease or a history of cardiac dysfunction or conduction abnormalities including any of the following:
    a. Unstable angina pectoris or documented myocardial infarction within 6 months prior to study entry.
    b. Symptomatic pericarditis.
    c. Documented congestive heart failure (CHF) (New York Heart Association [NYHA] Class III-IV).
    d. Left ventricular ejection fraction (LVEF) < 50% as determined by multigated acquisition (MUGA) scan or echocardiogram (ECHO).
    e. Ventricular arrhythmias except for benign premature ventricular contractions.
    f. Other cardiac arrhythmias requiring a pacemaker or not controlled with medication.
    g. Long QT syndrome (corrected QT interval by Fredericia [QTcF] > 450 ms, average of triplicate determinations at screening), or diagnosed or suspected long QT syndrome or known family history of long QT syndrome.
8. Clinically severe pulmonary compromise resulting from intercurrent pulmonary illnesses including, but not limited to, any underlying pulmonary disorder (i.e., pulmonary emboli within three months of the study enrolment, severe asthma, severe chronic obstructive pulmonary disease [COPD], restrictive lung disease, pleural effusion etc.), and any autoimmune, connective tissue or inflammatory

disorders with pulmonary involvement (i.e., rheumatoid arthritis, Sjogren's syndrome, sarcoidosis etc.), or prior pneumonectomy.

9. History of non-infectious interstitial lung disease (ILD)/pneumonitis that required steroids, has current ILD/pneumonitis, or has suspected ILD/pneumonitis that cannot be ruled out by imaging at screening.
10. Pregnant or lactating women.
11. Any serious medical condition or abnormality in clinical laboratory tests that, in the investigator's judgment, precludes the patient's safe participation in and completion of the study.
12. Current known infection with hepatitis B virus (HBV), or hepatitis C virus (HCV). Patients with past HBV infection or resolved HBV infection (defined as having a negative hepatitis B surface antigen [HBsAg] test and a positive hepatitis B core antibody [HBcAb] test, accompanied by a negative HBV DNA test) are eligible. Patients positive for HCV antibody are eligible only if polymerase chain reaction (PCR) is negative for HCV RNA.
13. Known human immunodeficiency virus (HIV) infection that is not well controlled. All of the following criteria are required to define an HIV infection that is well controlled: undetectable viral RNA, CD4-positive cells' count ≥350, no history of AIDS-defining opportunistic infection within the past 12 months, and stable for at least 4 weeks on the same anti-HIV medications (meaning there are no expected further changes in that time to the number or type of antiretroviral drugs in the regimen). If an HIV infection meets the above criteria, monitoring of viral RNA load and CD4-positive cells' count is recommended.
14. History of a major surgical procedure (defined as requiring general anesthesia) or significant traumatic injury within 21 days prior to randomization, or patients who have not recovered from the side effects of any major surgery.
15. History of uncontrolled seizures, CNS disorders or serious and/or unstable pre-existing psychiatric disability judged by the investigator to be clinically significant and adversely affecting compliance to study drugs or interfering with subject safety.
16. Patients requiring concomitant use of chronic systemic (intravenously [IV] or oral) corticosteroids at doses higher than 8 mg dexamethasone per day or other immunosuppressive medications except for managing adverse events (AEs), including immune-related adverse events (irAEs) for patients that received

immunotherapy in a previous line; (inhaled steroids or intra articular steroid injections are permitted in this study).

Note: The use of stable corticosteroid therapy in patients with brain metastases can be discussed with the Medical Monitor.

17. Patients with known substance abuse or any other medical conditions such as clinically significant cardiac or psychological conditions, that may, in the opinion of the investigator, interfere with the subject's participation in the clinical study or evaluation of the clinical study results.
18. Participants who are unable or unwilling to comply with the requirements of the protocol in the opinion of the investigator.
