# Supplementary material for: Patritumab deruxtecan in leptomeningeal metastatic disease of solid tumors: the phase 2 TUXEDO-3 trial
Source: Nat Med. 2025 May 30;31(8):2797–805. doi: 10.1038/s41591-025-03744-1 (PMC12353872; doi:10.1038/s41591-025-03744-1)
Supplement: Supplementary file 2 — Reporting Summary [file 41591_2025_3744_MOESM2_ESM.pdf]

nature portfolio

Corresponding author(s): Matthias Preusser

Last updated by author(s): Apr 25, 2025

Reporting Summary

Nature Portfolio wishes to improve the reproducibility of the work that we publish. This form provides structure for consistency and transparency in reporting. For further information on Nature Portfolio policies, see our Editorial Policies and the Editorial Policy Checklist.

Statistics

For all statistical analyses, confirm that the following items are present in the figure legend, table legend, main text, or Methods section.

n/a	Confirmed	
☐	☒	The exact sample size (*n*) for each experimental group/condition, given as a discrete number and unit of measurement
☐	☒	A statement on whether measurements were taken from distinct samples or whether the same sample was measured repeatedly
☐	☒	The statistical test(s) used AND whether they are one- or two-sided<br>*Only common tests should be described solely by name; describe more complex techniques in the Methods section.*
☐	☒	A description of all covariates tested
☐	☒	A description of any assumptions or corrections, such as tests of normality and adjustment for multiple comparisons
☐	☒	A full description of the statistical parameters including central tendency (e.g. means) or other basic estimates (e.g. regression coefficient) AND variation (e.g. standard deviation) or associated estimates of uncertainty (e.g. confidence intervals)
☐	☒	For null hypothesis testing, the test statistic (e.g. *F*, *t*, *r*) with confidence intervals, effect sizes, degrees of freedom and *P* value noted<br>*Give P values as exact values whenever suitable.*
☒	☐	For Bayesian analysis, information on the choice of priors and Markov chain Monte Carlo settings
☒	☐	For hierarchical and complex designs, identification of the appropriate level for tests and full reporting of outcomes
☒	☐	Estimates of effect sizes (e.g. Cohen's *d*, Pearson's *r*), indicating how they were calculated

Our web collection on statistics for biologists contains articles on many of the points above.

Software and code

Policy information about availability of computer code

Data collection	No software was used.
Data analysis	R software (version 4.3.2) within the RStudio environment (version 2023.12.1+402).

For manuscripts utilizing custom algorithms or software that are central to the research but not yet described in published literature, software must be made available to editors and reviewers. We strongly encourage code deposition in a community repository (e.g. GitHub). See the Nature Portfolio guidelines for submitting code & software for further information.

Data

Policy information about availability of data

All manuscripts must include a data availability statement. This statement should provide the following information, where applicable:

- Accession codes, unique identifiers, or web links for publicly available datasets
- A description of any restrictions on data availability
- For clinical datasets or third party data, please ensure that the statement adheres to our policy

Data collected within the TUXEDO-3 study will be made available to researchers upon reasonable request. Access to the data is controlled to ensure the protection of participant privacy and compliance with applicable data protection laws and regulations. Data will be shared upon revision and approval based on scientific merit by the TUXEDO-3 management group (which includes a qualified statistician) of a detailed proposal for their use. The data required for the approved, specified purposes and the trial protocol will be provided after the completion of a data-sharing agreement that will be set up by the study sponsor (MEDSIR). All data

provided will be anonymized to respect the privacy of patients who have participated in the trial. Estimate timeframe for response will be within 30 days. Please, address requests for data to the corresponding author (Dr. Matthias Preusser; matthias.preusser@meduniwien.ac.at).

Research involving human participants, their data, or biological material

Policy information about studies with human participants or human data. See also policy information about sex, gender (identity/presentation), and sexual orientation and race, ethnicity and racism.

Reporting on sex and gender	Female (N=20) and male (N=2) patients were included in this study
Reporting on race, ethnicity, or other socially relevant groupings	All patients were Caucasian, except for three individuals whose race was not reported, as it was not a mandatory requirement in data collection
Population characteristics	Male or female adult patients with histologically documented solid tumor of any type and treatment-naive LMD; no need for immediate local treatment; either type I LMD, defined by positive CSF cytology or leptomeningeal biopsy, or type II LMD, defined by clinical findings and neuroimaging only, according to Clinical Practice Guidelines by EANO-ESMO; life expectancy ≥6 weeks; LVEF ≥50% as per MUGA scan or echocardiogram, KPS ≥70%, ECOG PS 0-2. A total of 20 patients (18 female, 2 male) were included in the intention-to-treat population. Nine (45%) had type I and 11 (55%) had type II LMD, 14 (70%) had advanced disease at diagnosis, and the median number of previous treatment lines in the advanced setting was 2 (range 0-6). The primary tumor locations were breast in 12 (60%) patients, lung in 6 (30%) patients, melanoma in 1 (5%), and ovary in 1 (5%) patient. Previous treatment included ADCs (35%), tyrosine kinase inhibitors (20%) or chemotherapy combined with immunotherapy (20%). The median age at inclusion was 51.5 (range: 40–60) years, and 12 (60%) had neurological symptoms at baseline. Two (10%) patients did not have evaluable CNS lesions at baseline, and seven (35%) patients did not have evaluable extracranial lesions at baseline. Twelve participants (60%) had visceral disease, and 6 (30%) had brain-only disease.
Recruitment	Patients were recruited according to predefined inclusion criteria from seven sites across Austria and Spain. Eligible patients were identified by medical oncologists and were invited to participate in the TUXEDO-3 study during routine clinical visits. Participation was voluntary and written informed consent was obtained from all individuals prior to enrolment. Efforts were made to minimize possible selection bias by applying consistent eligibility criteria across all sites and by offering participation to all eligible patients.
Ethics oversight	The TUXEDO-3 study was conducted in accordance with the Declaration of Helsinki, the International Conference on Harmonization Good Clinical Practice guidelines and applicable regulations and laws from the recruiting countries, which were Austria and Spain. The study was approved by the ethics committee of the Instituto Valenciano de Oncología, Valencia (Spain). Written informed consent was obtained from each patient. None of the study participants received compensation for participation in the study.

Note that full information on the approval of the study protocol must also be provided in the manuscript.

Field-specific reporting

Please select the one below that is the best fit for your research. If you are not sure, read the appropriate sections before making your selection.

☒ Life sciences ☐ Behavioural & social sciences ☐ Ecological, evolutionary & environmental sciences

For a reference copy of the document with all sections, see nature.com/documents/nr-reporting-summary-flat.pdf

Life sciences study design

All studies must disclose on these points even when the disclosure is negative.

Sample size	We planned to assign 20 patients to this cohort. The protocol specified one interim analysis with 10 evaluable patients, based on Simon's two-stage design. The study would continue with the second stage if ≥1 patient alive after 3 months was observed. The critical value for the final analysis in this cohort was ≥3/20 patients alive after 3 months. The null hypothesis could have been rejected if ≥15% of patients were alive at 3 months. This design yielded a type I error rate of 10% and a power of 88% to reject the null hypothesis.
Data exclusions	Main exclusion criteria included previous systemic therapy with any anti-HER3 directed drug; treatment with approved or investigational cancer therapy within 14 days prior to initiation of study drug; concurrent malignancy or malignancy within 5 years of study enrollment with the exception of carcinoma in situ of the cervix, non-melanoma skin carcinoma, or stage I uterine cancer; CNS disorders; active cardiac disease or a history of cardiac dysfunction or conduction abnormalities within 6 months prior to study; current infection with hepatitis B virus, hepatitis C virus, or human immunodeficiency virus; major surgical procedure or significant traumatic injury within 21 days prior to randomization; participants who are unable or unwilling to comply with the requirements of the protocol.
Replication	This study is a prospective trial and thus no replication was foreseen within the scope of this study. Replication of the results requires further clinical trials
Randomization	This is a prospective, international, multicenter, single-arm phase II trial
Blinding	This is a prospective, international, multicenter, single-arm phase II trial

April 2023

Reporting for specific materials, systems and methods

We require information from authors about some types of materials, experimental systems and methods used in many studies. Here, indicate whether each material, system or method listed is relevant to your study. If you are not sure if a list item applies to your research, read the appropriate section before selecting a response.

Materials & experimental systems

n/a	Involved in the study
☐	☒ Antibodies
☒	☐ Eukaryotic cell lines
☒	☐ Palaeontology and archaeology
☒	☐ Animals and other organisms
☐	☒ Clinical data
☒	☐ Dual use research of concern
☒	☐ Plants

Methods

n/a	Involved in the study
☒	☐ ChIP-seq
☒	☐ Flow cytometry
☒	☐ MRI-based neuroimaging

Antibodies

Antibodies used

The expression of HER3 was determined using the primary antibody anti-HER3 SP438 clone (Roche Diagnostics). The expression of HER2, ER, and PgR was only determined in breast cancer patient samples using VENTANA anti-HER2/neu (4B5) (ref: 790-4493), CONFIRM anti-ER (SP1) (ref: 790-4324), and CONFIRM anti-PgR (1E2) (ref:790-2223) clones (all Roche Diagnostics), respectively. Antibody dilutions were performed following the manufacturer's instructions.

Validation

Antibody validation was performed following established guidelines to ensure specificity, sensitivity, and reproducibility. According to the manufacture's information, all antibodies have been validated to detect the corresponding receptors in formalin-fixed, paraffin-embedded tissue from human samples.

Clinical data

Policy information about clinical studies

All manuscripts should comply with the ICMJE guidelines for publication of clinical research and a completed CONSORT checklist must be included with all submissions.

Clinical trial registration

NCT05865990

Study protocol

The full trial protocol is included in the submission

Data collection

Recruitment and data collection were performed between January 2024 and July 2024 across seven sites in Autria (Medical University of Vienna and SCRI-CCCIT) and Spain (Hospital Arnau de Vilanova, Hospital Universitari Dexeus, Hospital Beata María Ana, Hospital Universitario Virgen del Rocío, and Hospital Universitari Vall D'Hebron).

Outcomes

The primary endpoint for this cohort was the 3-month OS rate, defined as the rate of patients alive at 3 months after the start of the study treatment. Secondary endpoints included investigator-assessed ORR as per RANO-BM for intracranial lesions and as per RECIST v.1.1 for extracranial and overall lesions; investigator-assessed CBR, DCR, TTR, DoR, PFS as per RANO-BM for intracranial lesions and RECIST v.1.1 for extracranial and overall lesions; safety and toxicity of HER3-DXd according to the NCI-CTCAE v.5.0; quality of life and neurocognitive function at cycles 1, 3, 5, 8 and end of treatment using the EORTC QoL questionnaire and the brain cancer specific questionnaire; and neurologic function at cycles 1, 3, 5, 8 and end of treatment using the NANO scale. Efficacy endpoints were analyzed according to HER3 expression levels in all patients. For those with breast cancer as the primary tumor, efficacy was also assessed based on ER, PgR and HER2 expression levels.

Plants

Seed stocks

N/A

Novel plant genotypes

N/A

Authentication

N/A
